# Hypoxia shapes the immune landscape in lung injury and promotes the persistence of inflammation

**DOI:** 10.1038/s41590-022-01216-z

**Published:** 2022-05-27

**Authors:** Ananda S. Mirchandani, Stephen J. Jenkins, Calum C. Bain, Manuel A. Sanchez-Garcia, Hannah Lawson, Patricia Coelho, Fiona Murphy, David M. Griffith, Ailiang Zhang, Tyler Morrison, Tony Ly, Simone Arienti, Pranvera Sadiku, Emily R. Watts, Rebecca. S. Dickinson, Leila Reyes, George Cooper, Sarah Clark, David Lewis, Van Kelly, Christos Spanos, Kathryn M. Musgrave, Liam Delaney, Isla Harper, Jonathan Scott, Nicholas J. Parkinson, Anthony J. Rostron, J. Kenneth Baillie, Sara Clohisey, Clare Pridans, Lara Campana, Philip Starkey Lewis, A. John Simpson, David H. Dockrell, Jürgen Schwarze, Nikhil Hirani, Peter J. Ratcliffe, Christopher W. Pugh, Kamil Kranc, Stuart J. Forbes, Moira K. B. Whyte, Sarah R. Walmsley

**Affiliations:** 1 University of Edinburgh Centre for Inflammation Research, Queen’s Medical Research Institute, University of Edinburgh, Edinburgh, UK; 2 Barts Cancer Institute, Queen Mary University of London, London, UK; 3 Wellcome Centre for Cell Biology, School of Biological Sciences, University of Edinburgh, Edinburgh, UK; 4 Intensive Care Unit, Royal Infirmary of Edinburgh, NHS Lothian, Edinburgh, UK; 5 Translational and Clinical Research Institute, Newcastle University, Newcastle upon Tyne, UK; 6 Department of Respiratory Medicine, Newcastle upon Tyne Hospitals NHS Foundation Trust, Newcastle upon Tyne, UK; 7 Roslin Institute, University of Edinburgh, Edinburgh, UK; 8 Centre for Regenerative Medicine, University of Edinburgh, Edinburgh, UK; 9 Nuffield Department of Medicine Research Building, Nuffield Department of Medicine, University of Oxford, Oxford, UK; 10 Ludwig Institute for Cancer Research, Nuffield Department of Medicine, University of Oxford, Oxford, UK; 11 The Francis Crick Institute, London, UK

**Keywords:** Monocytes and macrophages, Acute inflammation, Mucosal immunology, Haematopoiesis

## Abstract

Hypoxemia is a defining feature of acute respiratory distress syndrome (ARDS), an often-fatal complication of pulmonary or systemic inflammation, yet the resulting tissue hypoxia, and its impact on immune responses, is often neglected. In the present study, we have shown that ARDS patients were hypoxemic and monocytopenic within the first 48 h of ventilation. Monocytopenia was also observed in mouse models of hypoxic acute lung injury, in which hypoxemia drove the suppression of type I interferon signaling in the bone marrow. This impaired monopoiesis resulted in reduced accumulation of monocyte-derived macrophages and enhanced neutrophil-mediated inflammation in the lung. Administration of colony-stimulating factor 1 in mice with hypoxic lung injury rescued the monocytopenia, altered the phenotype of circulating monocytes, increased monocyte-derived macrophages in the lung and limited injury. Thus, tissue hypoxia altered the dynamics of the immune response to the detriment of the host and interventions to address the aberrant response offer new therapeutic strategies for ARDS.

## Abstract (web summary)

Mirchandani and colleagues show that tissue hypoxia alters the number and phenotype of circulating monocytes and their differentiation into lung macrophages, with important implications for the resolution of inflammation in the lung.

## Main

ARDS, a clinical syndrome defined by bilateral opacites on chest imaging denoting the presence of lung inflammation, blood hypoxemia with tissue hypoxia and the requirement for positive-pressure ventilation, has a mortality rate of up to 40%^1,2^. Despite decades of research, effective therapies for ARDS remain elusive, with much of the treatment research focusing on modulating the inflammatory injury, because its persistence is a poor prognostic indicator^3,4^. However, the effects of blood hypoxemia and the ensuing tissue hypoxia on the persistence of inflammation in ARDS have not been fully investigated.

Macrophages have a key role in driving tissue inflammation resolution. In mice, lung macrophages can be subdivided into SiglecF^+^ alveolar macrophages (AMs) and SiglecF^–^ interstitial macrophages (IMs), which inhabit distinct anatomical niches. Under homeostatic conditions, AMs largely self-renew^5,6^ whereas IMs require recruitment of circulating monocytes^7,8^. Evidence is emerging for key roles for IMs in inflammation regulation^9,10^ and repair^11^.

In the present study, we investigated whether hypoxemia associated with ARDS, including severe COVID-19 disease, affected the accumulation of lung monocyte-derived macrophages (MDMs) and whether this, in turn, impacted the resolution of inflammation. We found that patients with ARDS had clinical evidence of persistent hypoxemia despite ventilatory support and were profoundly monocytopenic during the first 48 h of ventilation, an observation replicated in mouse models of hypoxic acute lung injury (ALI). We further showed that systemic responses to tissue hypoxia suppressed type I interferon (IFN) signaling and fundamentally altered bone marrow hematopoiesis, with ensuing consequences for the phenotype and number of monocytes in the blood and accumulation of MDMs in the lung during ALI. This, in turn, led to the persistence of inflammation. Critically, targeting this pathway with the monocyte and macrophage growth factor colony-stimulating factor 1 (CSF-1) corrected these hypoxia-mediated changes and drove inflammation resolution.

## Results

### ARDS is characterized by monocytopenia and an altered immune phenotype

**Fig. 1:**
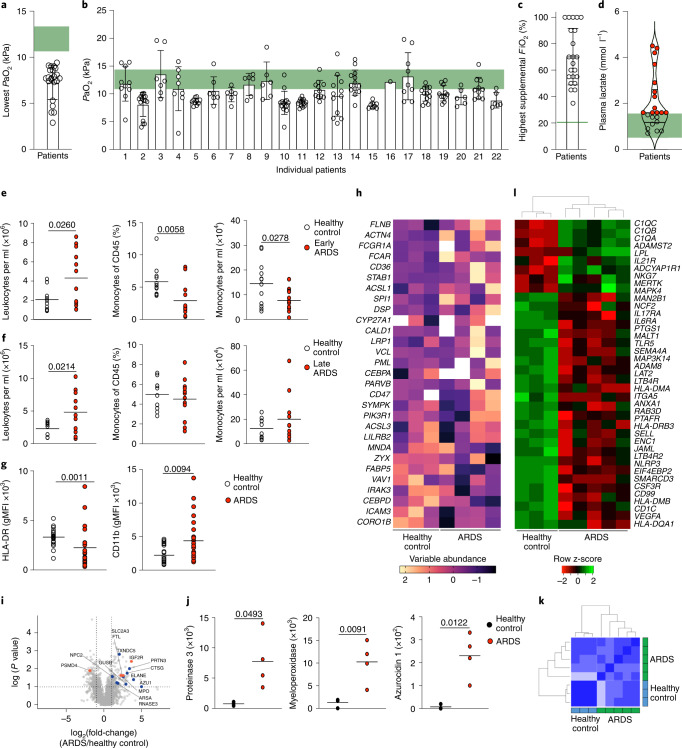
Patients with ARDS are monocytopenic early in the disease with phenotypically distinct circulating monocytes. **a**,**b**, Lowest (**a**) and all (**b**) partial pressures of oxygen (*P*aO_2_) from clinical arterial blood samples from ARDS patients, 24 h preceding research blood sampling (green: normal range). **c**,**d**, Highest recorded *F*iO_2_ (**c**) and highest recorded arterial plasma lactate level within 24 h of research sampling (**d**) in ARDS patients (red samples: lactate ≥upper limit of normal; green: normal local reference (0.5–1.6 mmol l^−1^)). **e**, Blood leukocyte counts, monocyte proportions and monocyte counts from ARDS patients, collected within 48 h of diagnosis (early ARDS) and a healthy volunteer cohort (HC). **f**, Blood leukocyte count, monocyte proportions and monocyte counts from ARDS patients collected between 48 h and 7 d (late ARDS) and HC. **g**, Monocyte HLA-DR and CD11b expression in HC and ARDS patients. **h**, CD14^++^CD16^−^ classical monocyte proteomic data from ARDS patients, relative to HC, for proteins associated with a human monocyte, in vitro hypoxic gene signature^13^. **i**, Classical (CD14^++^CD16^−^) monocyte proteome volcano plot from HC and ARDS patients. Significantly upregulated granule-associated proteins in ARDS patients versus HC (blue), a sample of known hypoxia-regulated proteins (orange). **j**, Classical monocytes proteinase 3, myeloperoxidase and azurocidin 1 copy numbers in HC and ARDS patients. **k**,**l**, Pearson’s correlation (**k**) and heatmap (**l**) of differentially expressed genes from HC and ARDS patient blood monocytes. Data in **a**–**c** are mean ± s.d. expressed as median (**e**,**f**) or shown as mean (**g**,**j**). In **a**,**c**–**g** and **j** each datapoint represents one patient/HC; in **b**, each datapoint represents one independent clinical sample. Statistical testing used was: unpaired, two-tailed Student’s *t*-test (**e**–**f** and **j**) and Mann–Whitney *U*-test (**g**).

**Extended Data Fig. 1:**
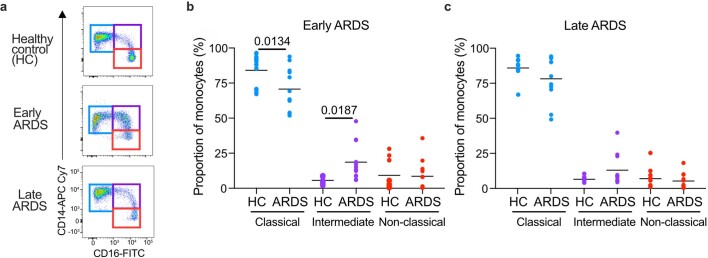
Monocyte sub-populations are altered early in ARDS. (**a**) Representative plots and proportions of monocyte sub-populations based on CD14 and CD16 expression early and (**b**) late (**c**). Each data point = one individual patient/ healthy donor control (HC), **b**, **c** Data+mean, one-way ANOVA with Holm-Sidak post-test.

Despite the heterogeneity of the etiologies leading to ARDS, one defining feature is blood hypoxemia and tissue hypoxia. We first characterized the arterial oxygen partial pressure (*P*aO_2_) in patients with moderate-to-severe ARDS. Patients were sampled within 1 week of a known insult or new or worsening respiratory symptoms in accordance with the Berlin criteria, namely, if they had bilateral opacities on a chest radiograph and evidence of respiratory failure with a *P*aO_2_/*F*iO_2_ (fraction inspired O_2_) < 200 mmHg and positive end-expiratory pressure >5 cmH_2_O. Patients with ARDS had a range of etiologies and associated pathogens (Table 1) and showed clinically important (Fig. 1a) and sustained (Fig. 1b) blood hypoxemia over a 24-h period despite supplementary oxygen therapy (Fig. 1c) and ventilatory support. In 13 of the 22 patients, this was associated with an elevated circulating lactate (Fig. 1d), indicating ongoing tissue hypoxia. To characterize the kinetics of circulating leukocyte populations, we sampled blood from ventilated patients <48 h from diagnosis of ARDS and commencement of positive-pressure ventilation (hereafter early ARDS) or 48 h to 7 d from diagnosis (late ARDS). Healthy donors were used as controls because tissue hypoxia is a common feature of critically unwell patients. Early ARDS patients had elevated circulating leukocyte counts (Fig. 1e), but significantly lower proportions and numbers of circulating monocytes (Fig. 1e) compared with controls. In late ARDS patients, circulating leukocyte numbers remained elevated compared with controls (Fig. 1f), but monocyte frequency and counts were equivalent (Fig. 1f). In early ARDS, we detected an increase in the proportion of CD14^+^CD16^+^ intermediate monocytes at the expense of classical CD14^++^CD16^−^ monocytes (Extended Data Fig. 1a–c)^12^. ARDS monocytes, irrespective of timepoint, had a lower expression of the major histocompatibility class II (MHC-II) marker HLA-DR (human leukocyte antigen-DR) and higher expression of CD11b compared with healthy controls (Fig. 1g). Proteomic analysis of sorted blood CD14^++^CD16^−^ ARDS monocytes indicated changes in the abundance of proteins with transcripts that had been reported to be sensitive to hypoxic culture in human monocytes^13^ when compared with healthy controls (Fig. 1h). We also observed a significant increase in secretory-granule content in ARDS CD14^++^CD16^−^ monocytes (Fig. 1i,j) which was associated with altered expression of hypoxia-regulated proteins, including SLC2A3^14^, IGFR2^15^, PSMD4^16^ and FTL^17^. NanoString analysis identified a specific transcriptional signature in ARDS monocytes (Fig. 1k), with 41 genes differentially expressed compared with healthy controls (Fig. 1l). Notably, four MHC complex genes (*HLA-DMA*, *HLA-DMB*, *HLA-DQA1* and *HLA-DRB3*), important for antigen-presenting function, as well as genes associated with monocyte adhesion and extravasation (*SELL*^18^), transendothelial migration (*CD99*^19^) and LPS signaling (*MAP2K4*^20^) and *MAP3K14*^21^) were significantly downregulated in ARDS monocytes (Fig. 1l). Thus, ARDS affected both the transcriptomic and the protein signature of blood monocyte

**Table 1:** ARDS patient cohort clinical characteristics and demographics

	Early ARDS (*n* = 11)	Late ARDS (*n* = 11)
Age (years)	58.8 (±11.5)	56.9 (±13.6)
Proportion females—number (%)	6 (55)	5 (45)
Body mass index (kg m^−2^)	35.8 (±11.6)	31.1 (±4.2)
APACHE2^a^ score	20.4 (±7.2)	18.8 (±8.6)
Pulmonary ARDS—number (%)	9 (82)	7 (64)
*Pulmonary aetiologies*^b^*—number (%)*		
Positive bacterial culture	4 (36)	4 (36)
Positive viral PCR^c^	2 (18)	1 (9)
Positive mycology	2 (18)	2 (18)
No positive microbiology samples	2 (18)	1 (9)
*Extrapulmonary aetiologies—number (%)*		
Fecal peritonitis	1 (9)	
Mediastinal soft-tissue infection	1 (9)	
Bacteremia		1 (9)
Biliary sepsis		1 (9)
Retropharyngeal abscess		1 (9)
Noninfective		1 (9)
*Index of tissue hypoxia*		
*Reference* P*aO*_2_*: 11.1–14.4* *kPa*^d^		
Lowest *P*aO_2_ in hospitalization preceding sampling (kPa)	4.38 (±1.72)	6.42 (±2.23)
Lowest *P*aO_2_ 24 h before sampling (kPa)	6.69 (±2.11)	8.15 (±1.90)
*Reference* F*iO*_*2*_: 21%		
Highest *F*iO_2_ in 24 h before sampling (%)	68.1 (±21.2)	72.3 (±22.3)
*Reference arterial lactate: 0.5–1.6* *mmol* *l*^*−1*^^d^		
Highest lactate within 24 h of sampling (mmol l^−1^)	2.45 (±1.28)	1.56 (±0.96)
*Receipt of organ-supportive therapies—number (%)*		
Invasive mechanical ventilation	7 (64)	7 (64)
Vasopressors	7 (64)	6 (55)
Renal replacement therapy	1 (9)	1 (9)
*Receipt of additional medications—number (%)*		
Dexamethasone^e^	0 (0)	0 (0)
Lopinavir or ritonavir	1 (9)	0 (0)
Tocilizumab	0 (0)	0 (0)
Hydroxychloroquine	1 (9)	1 (9)

Patient cohort and clinical characteristics demonstrate heterogeneity of etiology and evidence of clinically significant ongoing hypoxemia.

± data values refer to mean ± s.d.

^a^Acute Physiology and Chronic Health Evaluation Score 2 (APACHE2).

^b^One patient in each group returned mixed fungal and bacterial cultures, which could not be causatively differentiated.

^c^Two patients in the ‘Early’ group and one patient in the ‘Late’ group were SARS-CoV-2 positive.

^d^Reference ranges as indicated by the local health board (NHS Lothian).

^e^No other corticosteroids were administered.

### Experimental ARDS reproduces monocytopenia and phenotypic alterations

**Fig. 2:**
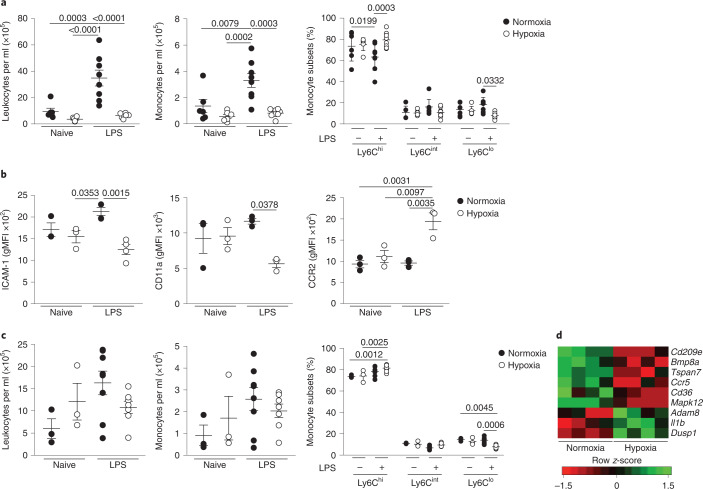
Hypoxic acute lung injury replicates early monocytopenia in mice and alters the circulating monocyte phenotype. **a**, Blood leukocyte counts, monocyte counts and proportion of blood monocyte subgroups in naive or LPS-treated mice housed in normoxia or hypoxia for 24 h. **b**, Classical monocyte (CD115^+^CD11b^+^Ly6C^hi^) surface expression of ICAM, CD11a and CCR2 at 24 h post-LPS. **c**, Blood leukocyte counts, monocyte counts and proportions of monocyte sub-populations in naive or LPS-treated mice housed in normoxia or hypoxia for 5 d post-LPS. **d**, Differentially expressed genes in circulating classical monocytes from LPS-treated mice housed in normoxia or hypoxia for 5 d. Data represent the mean ± s.e.m. Data for **a** and **c** are pooled from two independent experiments. b is representative of 2 experiments (*n*=3-4/ group). Each datapoint represents an individual mouse. Statistical testing: one-way ANOVA with Tukey’s multiple comparison test (**a** and **b**).

**Extended Data Fig. 2:**
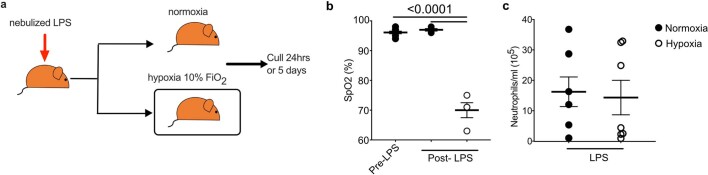
A hypoxic environment induces hypoxaemia with equivalent circulating neutrophil. (**a**) Schematic of normoxic and hypoxic LPS-induced ALI. (**b**) Oxygen saturations in mice were measured in mice at baseline pre-LPS nebulisation (pre-LPS) and 6 hours post-LPS (N LPS- mice housed in normoxia post-LPS, H LPS- mice housed in hypoxia post-LPS). (Pre-LPS n = 6, N LPS n = 3, H LPS n = 3). (**c**) Blood was collected from mice treated with LPS and placed in normoxia (N LPS) or hypoxia 10% (H LPS) for 5 days and circulating neutrophils quantified by flow cytometry (Live Singles CD45^+^Ly6G^+^CD11b^+^). b, c Each data point represents an individual mouse. Data shown as mean±SEM. c two pooled independent experiments. Statistical testing performed using one-way ANOVA with Tukey’s multiple comparisons test.

We next investigated whether the circulating monocyte profile alterations observed in ARDS patients were replicated by the induction of hypoxemia in a mouse model of ALI (Extended Data Fig. 2a). Mice exposed to 10% *F*iO_2_ demonstrated equivalent levels of hypoxemia to patients with ARDS (Extended Data Fig. 2b). Administration of nebulized lipopolysaccharide (LPS) induced an increase in circulating leukocytes and CD115^+^CD11b^+^ monocytes (Fig. 2a) in mice housed in normoxia for 24 h, relative to naive mice, yet this increase was absent in LPS-challenged mice housed in hypoxia (10% *F*iO_2_) immediately post-LPS for 24 h. A selective loss of nonclassical CD115^+^CD11b^+^Ly6C^lo^ monocytes in LPS-challenged hypoxic mice (Fig. 2a) relative to normoxic LPS-challenged mice was observed. Blood CD115^+^CD11b^+^Ly6C^hi^ classical monocytes from hypoxic LPS-challenged mice had decreased expression of the adhesion molecules intercellular adhesion molecule (ICAM)-1 and CD11a and increased expression of the CCR2 chemokine receptor, compared with normoxic counterparts (Fig. 2b). The absolute number of circulating leukocytes, CD115^+^CD11b^+^ monocytes (Fig. 2c) and Ly6G^+^CD11b^+^ neutrophils (Extended Data Fig. 2c) in the blood of normoxic or hypoxic LPS-treated mice normalized at day 5, although the proportion of Ly6C^lo^ monocytes remained contracted in hypoxic LPS-challenged mice compared with normoxic LPS-challenged controls (Fig. 2c). We also observed persistent alterations in the transcriptome of Ly6C^hi^ monocytes in hypoxic LPS-challenged mice (Fig. 2d), including decreased expression of the chemokine receptor *Ccr5* and the scavenger receptor *Cd36*, which are markers of monocyte maturity^22^ and increased expression of *Il1b*, an inflammatory cytokine associated with poor outcomes in ARDS^23^. Thus, in mice with ALI, hypoxia drives alterations in monocyte numbers and phenotype that parallel those described in ARDS patients.

### Tissue hypoxia prevents accumulation of lung MDMs

**Fig. 3:**
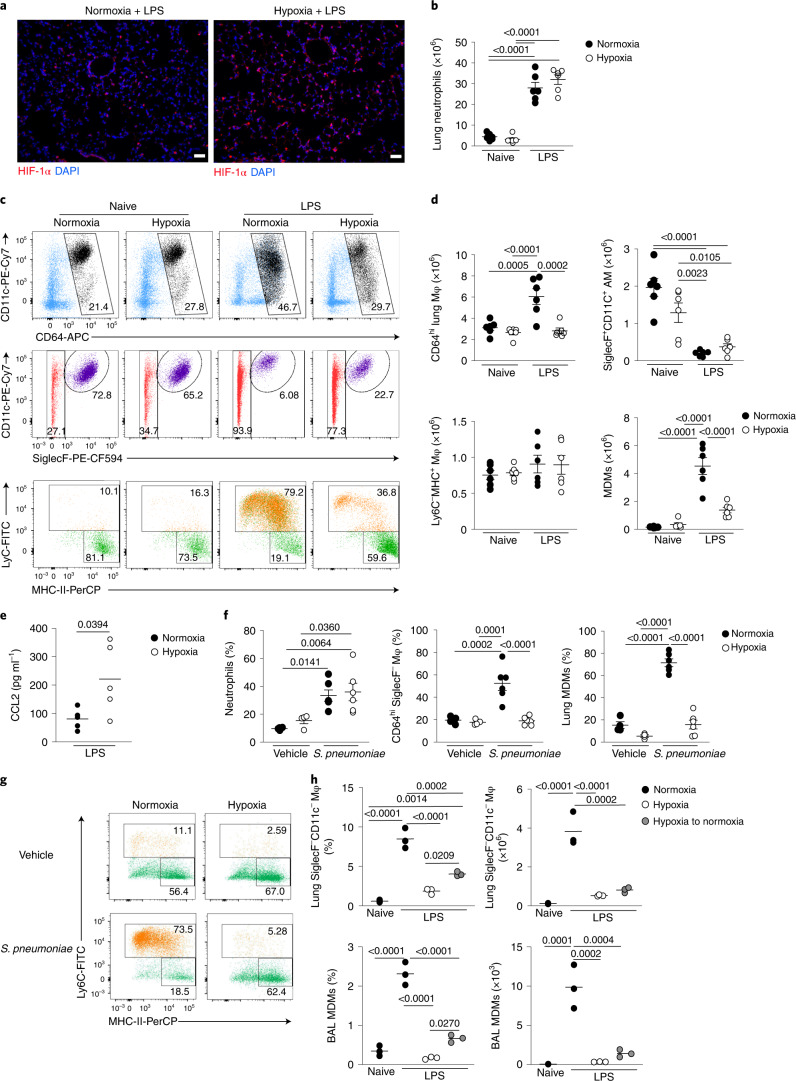
Systemic hypoxia hampers expansion of the CD64^hi^SiglecF^−^ macrophage niche in ALI and *S. pneumoniae* infection. **a**, Representative lung immunofluorescence HIF-1α and DAPI expression from LPS-challenged mice, housed in normoxia or hypoxia for 24 h. Scale bar, 50 μm. **b**, Absolute numbers of live lung neutrophils in naive or LPS-challenged mice housed in normoxia (N) or hypoxia (H) for 24 h (*n* = 6 per group). **c**, Representative dot plots of the CD64^hi^ macrophage compartment (top), CD64^hi^SiglecF^+^CD11c^+^ AMs (middle) and Ly6C and MHC-II expression by CD64^hi^SiglecF^−^ macrophages (bottom) in mice as in **b**. **d**, Absolute number of CD64^hi^ macrophages (Mφ), CD64^hi^SiglecF^+^CD11c^+^ AMs, Ly6C^−^MHC-II^+^ lung macrophages and CD64^hi^SiglecF^−^Ly6C^+^ MDMs as in **b**. **e**, BAL CCL2 levels from LPS-challenged mice housed in normoxia or hypoxia for 24 h. **f**, Frequencies of neutrophils among total lung leukocytes, of CD64^hi^SiglecF^−^ macrophages among lung CD64^hi^ macrophages and MDMs among CD64^hi^SiglecF^−^ macrophages in mice inoculated with *S. pneumoniae* (*n* = 6 per group) or vehicle control (Veh, *n* = 4 per group) and housed in normoxia or hypoxia until 24 h post-inoculation. **g**, Representative plots of CD64^hi^SiglecF^−^ macrophages in mice as in **f**. **h**, Frequency of lung CD64^hi^SiglecF^−^ macrophages among total leukocytes, absolute numbers of lung CD64^hi^SiglecF^−^ macrophages, proportion of BAL MDMs and absolute numbers of BAL MDMs in naive or LPS-challenged mice housed either in normoxia or hypoxia for 48 h or for 24 h in hypoxia, followed by 24 h of normoxia (hypoxia to normoxia) (*n* = 3 per group). Data represent the mean ± s.e.m. Data in **a** represent *n* = 3 per group; data in **b**–**e** are pooled from two independent experiments; data in **g** represent two independent experiments. Each datapoint represents an individual mouse. Statistical testing for **b**,**d**,**f** and **h** is by one-way ANOVA with Tukey’s multiple comparison test and for **e** by unpaired, two-tailed Student’s *t*-test.

**Extended Data Fig. 3:**
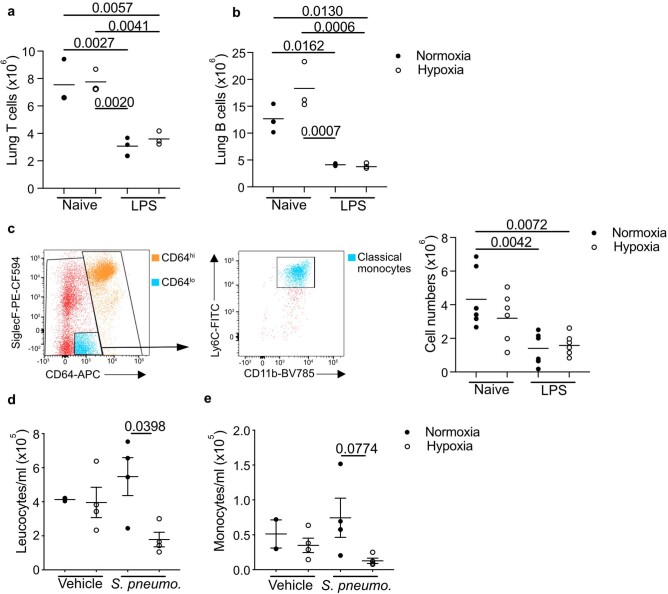
T cell and B cells are equivalent post-LPS and *Streptococcus pneumoniae* infection in hypoxia leads to leukopenia and monocytopenia. T cells (**a**) (Live, singles, CD45^+^, Lin^+^ (CD3/CD19/Ly6G) MHCII^−^ CD11b^−^) and B cells (**b**) (Live, singles, CD45^+^, Lin^+^ (CD3/CD19/Ly6G) MHCII^+^ CD11b^−^) were quantified in lung digests from mice housed in normoxia (N) or 10% FiO2 hypoxia (H) for 24 hours, left naïve or nebulised with LPS. Mice were inoculated with *Streptococcus pneumoniae* (Strep) or vehicle (Veh) intratracheally (i.t.) and housed in normoxia (N) or hypoxia (H) until 24 hours post-i.t. (**c)** Representative dot plots of gating strategy for classical monocytes in the lung gated on Singles Live CD45 + Lin- lung cells and associated counts in the lung 24 hours post-LPS challenge and housed in normoxia (N) and hypoxia (H). Blood cell counts and (c) monocyte counts mice challenged with vehicle or *strep pneumoniae* and housed in normoxia (N) or hypoxia (H) for 24 hours. Each point represents and individual mouse. Data shown as mean±SEM. Statistical testing performed using one-way ANOVA with Tukey’s multiple comparisons test.

Next we explored the effect of hypoxemia on LPS-induced inflammation in the lung. Consistent with tissue hypoxia, hypoxia-inducible factor 1α (HIF-1α) protein was most highly expressed in the lungs of hypoxic LPS-treated mice compared with normoxic LPS controls (Fig. 3a). LPS challenge significantly increased lung Ly6G^+^CD11b^+^ neutrophil numbers in hypoxia- or normoxia-housed mice compared with naive controls (Fig. 3b), with equivalent numbers of CD3^+^CD19^−^MHC-II^−^ T cells and CD3^−^CD19^+^MHC-II^+^ B cells in the lungs of these mice (Extended Data Fig. 3a,b). LPS challenge led to a significant expansion of the Lin^–^CD45^+^CD64^hi^ macrophage compartment in normoxia- but not hypoxia-housed mice (Fig. 3c,d). AMs are CD64^hi^SiglecF^+^CD11c^+^, whereas CD64^hi^SiglecF^–^ macrophages almost exclusively comprise IMs in health, and include inflammation-elicited, parenchymal or alveoli-localized, CD64^hi^SiglecF^–^Ly6C^+^ MDMs after injury. The number of CD64^hi^SiglecF^+^CD11c^+^ macrophages was equivalent in LPS-challenged mice housed in hypoxia or normoxia (Fig. 3c,d). Hypoxia significantly blunted the LPS-mediated expansion of the CD64^hi^SiglecF^–^ macrophage compartment observed in normoxic mice (Fig. 3c,d), an effect that appeared to be entirely attributable to the absence of CD64^hi^SiglecF^–^Ly6C^+^ MDMs in LPS-treated hypoxic mice (Fig. 3c,d). The number of CD64^hi^SiglecF^–^Ly6C^−^MHC-II^+^ macrophages was similar in all treatments (Fig. 3c,d). The reduction in the number of CD64^hi^SiglecF^–^Ly6C^+^ MDMs occurred despite elevated amounts of the monocyte chemoattractant CCL2 in the alveoli of LPS-treated hypoxic mice (Fig. 3e), and similar numbers of CD64^lo^CD11b^+^Ly6C^+^ monocytes in the lungs of hypoxic and normoxic LPS-treated mice (Extended Data Fig. 3c). These observations suggested that monocytes recruited to the lung after LPS treatment did not convert to CD64^hi^SiglecF^–^Ly6C^+^ MDMs in hypoxia. The contribution from intravascular cells to lung cell counts could not be completely excluded from these data.

To examine the effects of hypoxia on monocyte–macrophage dynamics in a model of severe streptococcal pneumonia, C57/BL6J mice were inoculated with D39 *Streptococcus pneumoniae* or vehicle and housed in either hypoxia or normoxia after a 4-h recovery period. Reduced blood leukocyte and monocyte counts were detected in infected mice housed in hypoxia compared with normoxia (Extended Data Fig. 3d,e). Although the number of lung neutrophils was equivalent in hypoxic and normoxic *S. pneumoniae*-infected mice (Fig. 3f), the accumulation of CD64^hi^SiglecF^–^ macrophages was reduced (Fig. 3f), with a particular absence of CD64^hi^SiglecF^–^Ly6C^+^ MDMs (Fig. 3f,g).

To determine whether hypoxia altered the monocyte–macrophage lung compartment directly, we returned LPS-challenged hypoxic mice to normoxic conditions, for a further 24 h, after 24 h of hypoxia. Mice returned to normoxia showed a significant increase in the proportion and number of CD64^hi^SiglecF^–^ macrophages and bronchoalveolar lavage (BAL) MDMs, compared with mice that remained in hypoxia (Fig. 3h). Together, these data indicated that hypoxia directly induced sustained changes in the lung macrophage compartment during various inflammatory challenges.

### Hypoxia directly alters hematopoiesis

**Fig. 4:**
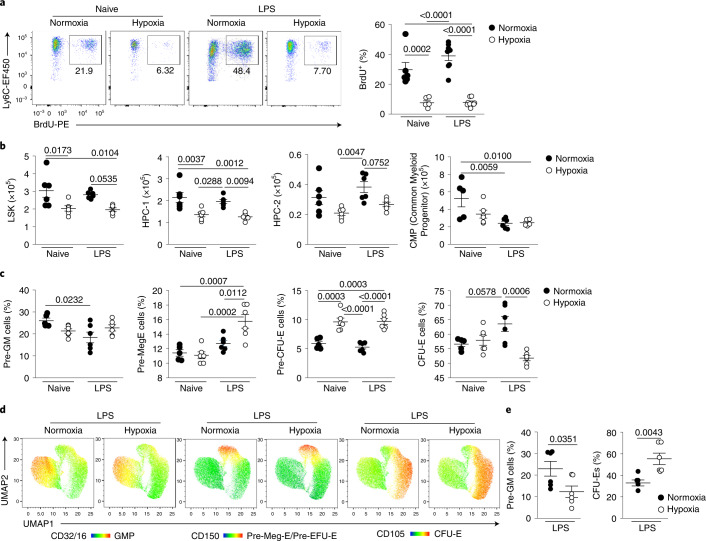
Systemic hypoxia alters BM hematopoiesis toward increased erythropoiesis. **a**, Representative dot plots gated on live CD45^+^Lin^−^(CD3/CD19/Ly6G)CD115^+^Ly6C^hi^ cells and proportion of BrdU^+^ monocytes in naive or LPS-challenged mice housed in normoxia or hypoxia for 24 h and pulsed with BrdU for the last 12 h (naive, *n* = 5–6; LPS treated, *n* = 8 in). **b**, Absolute numbers of BM LSK cells, CD48^+^CD150^−^ HPC-1, CD48^+^CD150^+^ HPC-2 (*n* = 6 per group) and Lin^−^cKit^+^Sca1^−^CD127^−^CD16/32^−^CD34^+^ CMPs (*n* = 5–6 group) in mice as in **a**. **c**, Proportion of BM pre-GMs, pre-Meg-E, pre-CFU-E and CFU-E CD41^−^CD32/16^−^ cells in mice as in **a** (*n* = 6 per group). **d**,**e**, Representative UMAP analysis of BM cells gated on live CD45^+^Lineage^−^Sca1^−^C-Kit^+^CD41^−^CD32/16^−^ cells (**d**) and summary data of proportions of pre-GM and CFU-E cells (**e**) measured in the BM of mice treated with LPS and housed in normoxia (N) or hypoxia (H) for 5 d (*n* = 6 per group). Data are shown as the mean ± s.e.m. Each datapoint represents an individual mouse. Data in **a**–**e** are pooled from two independent experiments. Statistical testing: for **a**–**c** is by one-way ANOVA with Tukey’s multiple comparison test and for **e** by unpaired, two-tailed Student’s *t*-test.

**Extended Data Fig. 4:**
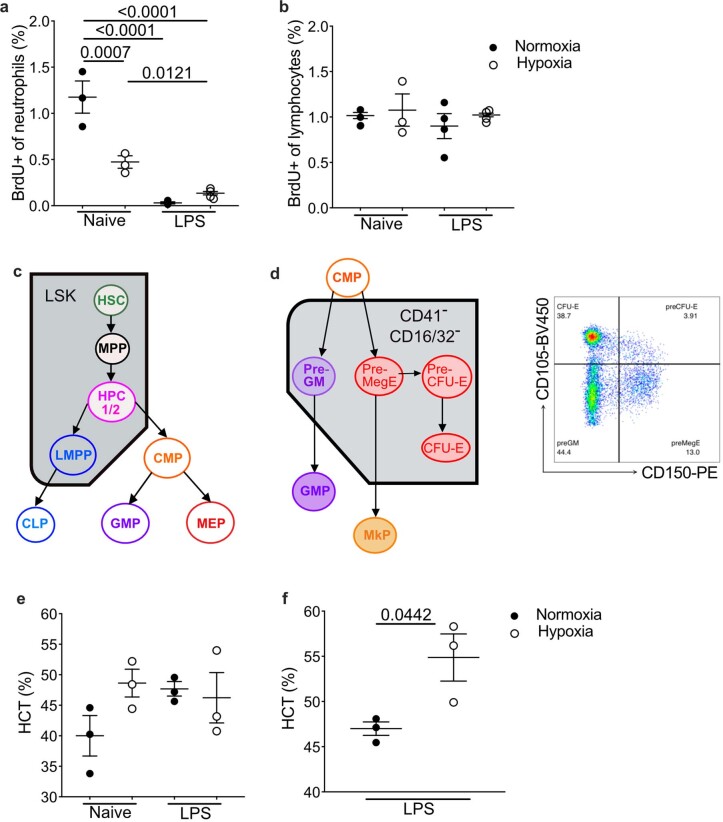
Impact of hypoxia on bone marrow cell egress and composition. (**a**) BrdU+ blood lymphocytes (CD3 and CD19^+^) and (**b**) BrdU+ blood neutrophil proportion in mice treated with LPS and housed in normoxia (N) and hypoxia (H) for 24 hours (Naïve N and H n = 3, LPS N and H n = 4). Data representative of 2 experiments (**c**) Schematic showing hematopoietic hierarchy with Lin^−^Sca^−^C-Kit^+^ (LSK) compartment and progenitors (HSC-hematopoietic stem cell, MPP- multipotent progenitor, HPC- hematopoietic progenitors, CMP- common myeloid progenitor, LMPP- lymphoid-primed multipotent progenitor, CLP- common lymphoid progenitor, GMP- granulocyte/monocyte progenitor, MEP-Megakaryocyte/erythrocyte progenitor). (**d**) Schematic showing erythrocytosis and monopoiesis (Pre-GM-pre-granulocyte/monocyte precursor, Pre-MegE - megakaryocyte-erythrocyte precursor, Pre-CFU-E - pre-colony forming unit erythroid, CFU-E - Colony forming unit erythroid, GMP – granulocyte-monocyte precursor, MkP- megakaryocyte precursor) gating strategy for bone marrow common myeloid progenitor progeny on CD41^−^CD16/32^−^ cells. (**e**) Blood hematocrit at 24 hours (n = 3/ group, data representative of 2 experiments) or (f) 5 days in mice treated with LPS and housed in normoxia (N) and hypoxia (H) (n = 3/group, data representative of 2 experiments) was measured. a, b Mean±SD. Statistical testing performed using one-way ANOVA with Tukey’s multiple comparisons test, e, f Mean±SD. f, statistical testing performed using unpaired two-sided t-test.

To determine the mechanism by which hypoxia regulated the number of circulating monocytes, we measured bone marrow (BM) output by pulsing naive or LPS-challenged mice, housed in hypoxia or normoxia, with bromodeoxyuridine (BrdU) 12 h post-LPS^12,24^. Hypoxemic LPS-challenged mice had a 80% reduction in the proportion of BrdU^+^CD115^+^CD11b^+^Ly6C^hi^ monocytes compared with normoxic LPS-challenged counterparts (Fig. 4a). LPS equally reduced the proportion of blood BrdU^+^ neutrophils in hypoxic and normoxic mice compared with naive controls (Extended Data Fig. 4a), with a similar frequency of BrdU^+^ lymphocytes in all samples (Extended Data Fig. 4b). Examination of the BM stem cell compartment (Extended Data Fig. 4c) 24 h post-hypoxic exposure indicated a reduction in absolute numbers of Lin^–^Sca-1^+^Kit^+^ (LSK) cells in hypoxic mice compared with normoxic mice independent of LPS treatment (Fig. 4b), with a specific reduction in the CD48^+^CD150^−^ HPC-1 and CD48^+^CD150^+^ HPC-2 hematopoietic progenitor cells (HPCs; Fig. 4b), which have restricted multipotency^25^. Irrespective of oxygenation, LPS treatment reduced the absolute number of Lin^−^cKit^+^Sca1^−^CD127^−^CD16/32^−^CD34^+^ common myeloid progenitor cells (CMPs) compared with naive control mice (Fig. 4b).

As erythrocytes and monocytes originate from CMPs^26^ (Extended Data Fig. 4d), we investigated whether hypoxia shifted hematopoiesis in favor of red blood cell (RBC) production by measuring the effect of hypoxia on CMP progeny (Lin^−^cKit^+^CD41^−^CD32/16^−^) 24 h after LPS challenge^26^. Hypoxia did not affect the number of CD150^−^CD105^−^ pre-granulocyte/monocyte precursors (pre-GMs) (Fig. 4c), but significantly increased the proportion of CD150^+^CD105^−^ megakaryocyte-erythroid precursor (pre-Meg-E) cells (Fig. 4c), and led to an increase in downstream CD150^+^CD105^+^ pre-colony-forming unit erythroid precursor (pre-CFU-E) cells (Fig. 4c). Although hematocrit values were equivalent at this 24-h timepoint (Extended Data Fig. 4e), BM CFU-E cell proportions were reduced in LPS-treated hypoxic mice compared with normoxic counterparts (Fig. 4c), suggesting increased red cell egress. The hematocrit of LPS-treated hypoxic mice was increased compared with normoxic controls at day 5 (Extended Data Fig. 4f). UMAP (Uniform Manifold Approximation and Projection) analysis of Lin^−^cKit^+^CD41^−^CD32/16^−^ cells within the BM compartment post-LPS indicated that skewing of the CMPs toward erythropoiesis was achieved by day 5 (Fig. 4d,e). Taken together, these data demonstrated that hypoxia altered hematopoiesis, reducing monocyte BM output.

### Hypoxia suppresses type I IFN signaling

**Fig. 5:**
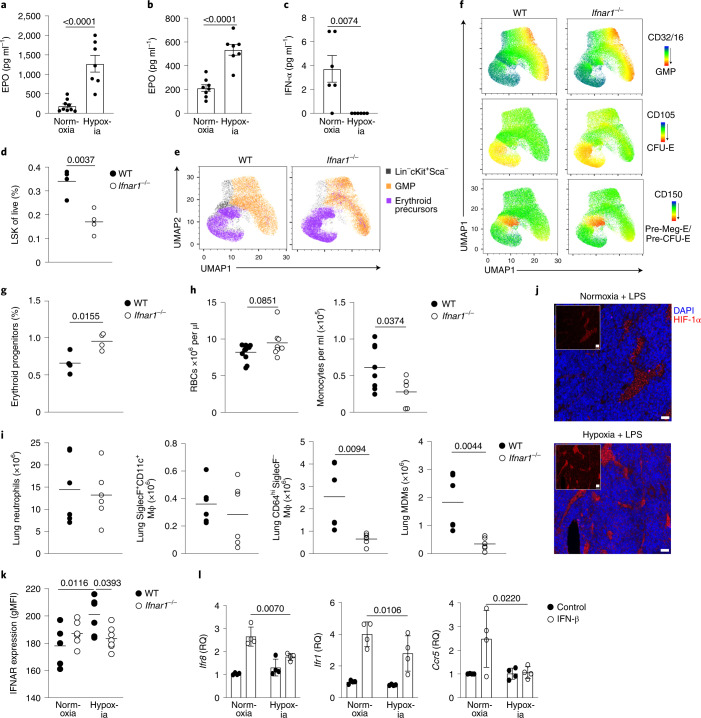
Hypoxia regulates type I IFN responses, hindering lung CD64^hi^SiglecF^−^ macrophage expansion in response to LPS. **a**,**b**, Serum EPO levels in mice challenged with LPS and housed in normoxia or hypoxia for 24 h (*n* = 9 normoxia, *n* = 7 hypoxia) (**a**) or 5 d (*n* = 7 per group) (**b**). **c**, Serum IFN-α in in mice challenged with LPS and housed in normoxia or hypoxia for 24 h (*n* = 6 per group). **d**, Proportion of LSK cells in the BM of WT or *Ifnar1*^−/−^ mice 24 h post-LPS challenge (*n* = 4 per group). **e**, Manual gating of erythroid precursors and granulocyte/macrophage progenitors (GMPs) in LSK cells displayed on UMAP projection in mice as in **d**. **f**, Representative expression of CD32/16 (GMP marker) and CD150/CD105 (erythroid progenitor-associated markers) in LSK cells from mice as in **d** using the Pronk gating strategy^26^ displayed on UMAP projection. **g**, Proportion of erythroid progenitor cells (combined MEPs, pre-CFU-E and CFU-E cells) in WT and *Ifnar1*^−/−^ BM 24 h post-LPS (*n* = 4 per group). **h**, Peripheral RBCs (*n* = 10 WT, *n* = 8 knockout) and monocyte counts at day 5 post-LPS in WT and *Ifnar1*^−/−^ mice. **i**, Neutrophils, CD64^hi^SiglecF^+^CD11c^+^ macrophages, CD64^hi^SiglecF^−^ macrophages and MDM numbers in the lungs of WT and *Ifnar1*^−/−^ mice 24 h post-LPS. **j**, Representative HIF-1α and DAPI expression in the femoral BM in mice challenged with LPS and housed in normoxia (N LPS) or hypoxia (H LPS) for 24 h. Scale bar, 20 μm. **k**, IFNAR expression in the BM LSK in naive (*n* = 5–6 per group) or LPS-treated mice (*n* = 6 per group) housed in normoxia or hypoxia for 24 h. **l**, Fold change in Quantitative PCR of *Irf8*, *Irf1* and *Ccr5* expression (normalized to actin-β, relative quantification) in BM cells from naive mice cultured in normoxia or hypoxia for 4 h ± IFN-β (*n* = 3 per group) relative to untreated normoxia control. Data represent the mean ± s.e.m. All datapoints represent individual mice. Statistical testing for **a–d** and **g**–**i** was by unpaired, two-sided Student’s *t*-test, for **k** by one-way ANOVA with Tukey’s multiple comparison test and for **l** by two-way ANOVA with Šídák's multiple comparison post-test. The data in **a**–**c** and **d**–**i** represent two independent experiments, and represent *n* = 3 per group in **j** and two independent pooled experiments in **l**.

**Extended Data Fig. 5:**
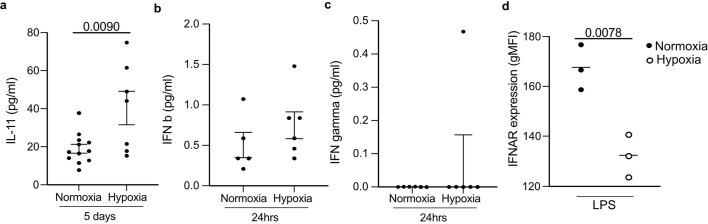
Hypoxia elevates circulating IL-11 levels without altering IFN beta and IFN gamma levels and reducing IFNAR expression in circulating classical monocytes. (**a**) Serum IL-11, (**b**) IFN beta and (**c**) IFN gamma level mice treated with nebulised LPS and housed in normoxia (N) or hypoxia (H), for 24 hours or 5 days (as indicated on figure), were measured by ELISA as per manufacturers’ instructions. Data mean±SEM. Each data point represents an individual mouse. (**d**) IFNAR expression was measured by flow cytometry in classical blood monocytes in mice treated with nebulized LPS and housed in either normoxia (N LPS) or hypoxia (H LPS) for 24 hours. **a**, **d**, Statistical testing unpaired two-sided t-test.

Next, we investigated the mechanism by which hypoxia suppressed monopoiesis in mice with ALI. Erythropoietin (EPO), an RBC growth factor, was significantly increased at 24 h (Fig. 5a) and day 5 post-LPS in hypoxia-housed compared with normoxia-housed mice (Fig. 5b). Interleukin (IL)-11, a hypoxia-responsive megakaryocyte and hematopoietic growth factor^27,28^, was also increased at day 5 post-LPS in hypoxic compared with normoxic mice (Extended Data Fig. 5a). Type I IFN (IFN-α and IFN-β) and type II IFN (IFN-γ) are known drivers of emergency monopoeisis^29,30^. Although IFN-β and IFN-γ were equivalent in hypoxic and normoxic LPS-treated mice, IFN-α was markedly reduced in hypoxic mice 24 h post-LPS challenge compared with normoxic counterparts (Fig. 5c and Extended Data Fig. 5b,c). *Ifnar1*^*–/–*^ mice, which lack the type I IFN receptor, had a contraction of the LSK compartment (Fig. 5d) and increased proportion of the megakaryocyte/erythrocyte progenitor (MEP)/pre-CFU-E/CFU-E erythroid progenitors 24 h after LPS treatment during normoxia (Fig. 5e–g). Then, 5 d post-LPS challenge, normoxic *Ifnar1*^*–/–*^ mice had enhanced numbers of circulating RBCs and were significantly monocytopenic compared with wild-type (WT) controls (Fig. 5h). In addition, normoxic *Ifnar1*^*–/–*^ mice had similar numbers of neutrophils and CD64^hi^SiglecF^+^ macrophages (Fig. 5i), but reduced numbers of CD64^hi^SiglecF^–^ macrophages and MDMs (Fig. 5i) in the lung 24 h post-LPS, compared with WT controls.

Increased HIF-1α stabilization was observed in the BM of hypoxic, LPS-treated, mice compared with normoxic counterparts (Fig. 5j), in keeping with tissue hypoxia. As hypoxia can alter type I IFN signaling in cancer^31^, we next investigated interferon-α/β receptor (IFNAR) expression in the BM. LSK cells from hypoxic mice 24 h post-LPS treatment did not upregulate IFNAR expression, compared with normoxic mice (Fig. 5k). IFNAR expression was also reduced in blood CD115^+^CD11b^+^Ly6C^hi^ monocytes from hypoxic LPS-challenged mice compared with normoxic counterparts (Extended Data Fig. 5d). BM cells from naive mice cultured in vitro, in hypoxia (1% O_2_), showed significant blunting of type I IFN-mediated *Irf*8, *Irf1* and *Ccr5* expression (Fig. 5l). Taken together, these findings suggested that hypoxia directly altered the immune response through local and systemic mechanisms.

### Loss of monocyte recruitment associated with persistence of inflammation

**Fig. 6:**
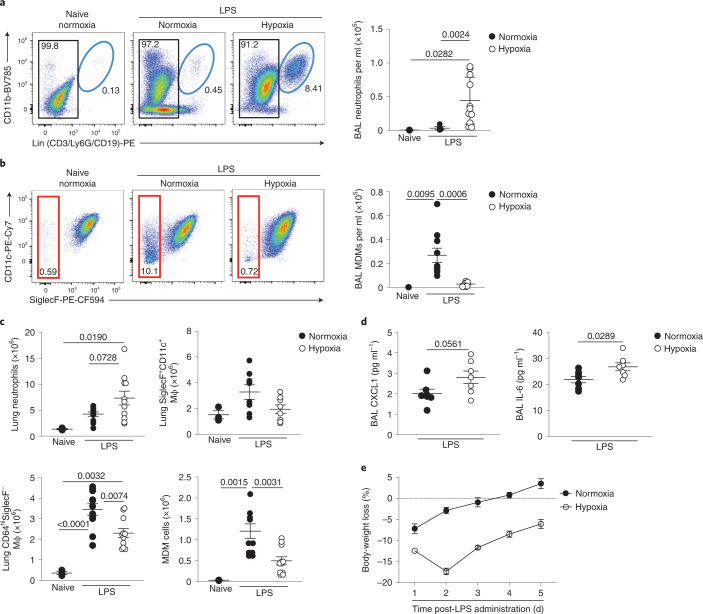
Ongoing CD64^hi^SiglecF^−^ macrophage expansion failure is associated with inflammation persistence in hypoxic ALI. **a**,**b**, Representative dot plots and absolute numbers of BAL neutrophils (**a**) and CD45^+^Ly6G^−^CD64^hi^SiglecF^−^ MDMs gated on CD45^hi^ cells (**b**) in mice treated with LPS and housed in normoxia or hypoxia for 5 d. **c**, Lung neutrophils, CD64^hi^SiglecF^+^CD11c^+^ macrophages, CD64^hi^SiglecF^−^ macrophages and CD64^hi^SiglecF^−^Ly6C^+^ MDM numbers in LPS-challenged mice housed in normoxia or hypoxia for 5 d. **d**, BAL CXCL1 and IL-6 levels in mice treated with LPS and housed in normoxia (N) or hypoxia (H) for 5 d (*n* = 6 N LPS, *n* = 7 H LPS). **e**, Daily weight changes from baseline in LPS-challenged mice housed in normoxia or hypoxia for 5 d (*n* = 4 per group). Data are shown as the mean ± s.e.m. Each datapoint represents an individual mouse. Statistical testing for **a**–**c** was by one-way ANOVA with Tukey’s multiple comparison test and for **d** by unpaired, two-tailed Student’s *t*-test. Data in **a**–**c** were pooled from three independent experiments and in **d** from two independent experiments.

We subsequently investigated the longer-term effects of systemic hypoxia on the myeloid compartment and inflammation resolution. Infiltrating neutrophils promote vascular injury, protein leak and alveolar epithelial damage in ARDS, and drive deleterious inflammatory responses in murine models of hypoxic ALI^32,33^. 5 d after LPS challenge, normoxic mice had very few neutrophils within the bronchoalveolar space whereas hypoxic mice showed evidence of ongoing inflammation with significant bronchoalveolar neutrophilia (Fig. 6a). Hypoxic LPS-treated mice had a reduction in the number of bronchoalveolar CD64^hi^SiglecF^–^ MDMs compared with normoxic counterparts (Fig. 6b). The total number of lung Ly6G^+^CD11b^+^ neutrophils was greater in hypoxic compared with normoxic mice at day 5 post-LPS (Fig. 6c) and, although the number of CD64^hi^SiglecF^+^CD11c^+^ macrophages had returned to baseline (Fig. 6c), the non-AM CD64^hi^SiglecF^–^ macrophages remained contracted (Fig. 6c), largely as a consequence of fewer CD64^hi^SiglecF^–^Ly6C^+^ MDMs (Fig. 6c). In addition, the BAL from hypoxic mice at this timepoint had higher CXCL1 and IL-6 (Fig. 6d), parameters reported to be elevated in ARDS nonsurvivors^23^. Moreover, hypoxic mice showed more sustained weight loss at day 5 post-LPS, compared with the normoxia-housed controls (Fig. 6e). Collectively, these data indicated that hypoxia-induced monocytopenia was associated with persistent lung inflammation.

### CSF-1 accelerates the resolution of lung inflammation in hypoxia

**Fig. 7:**
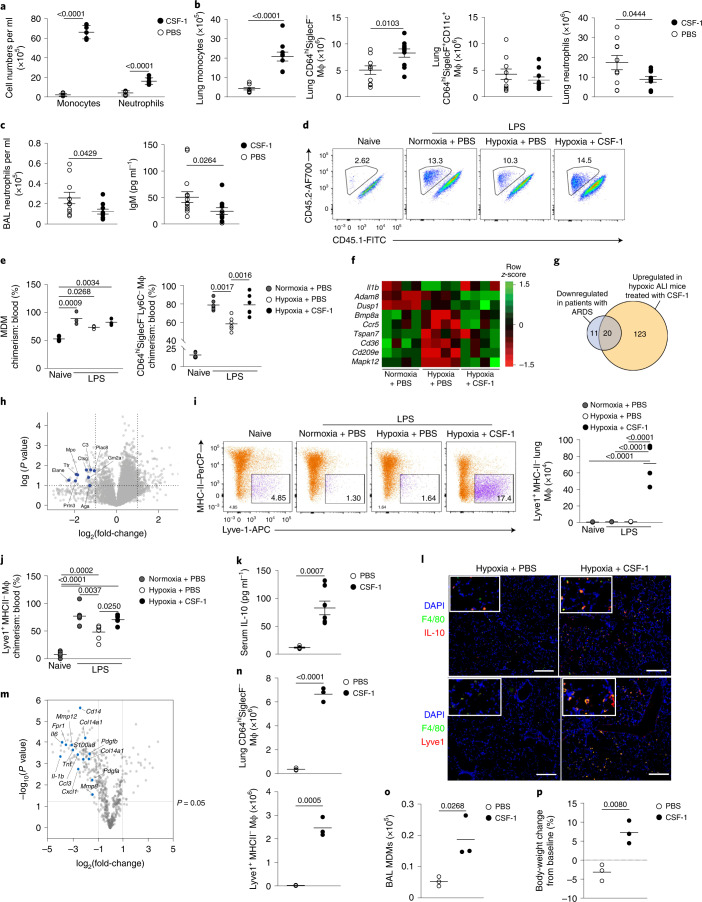
CSF-1 rescues the hypoxic monocytopenia driving inflammation resolution. **a**–**c**, Monocyte and neutrophil counts in the blood (**a**), monocyte, CD64^hi^SiglecF^−^ macrophage, CD64^hi^SiglecF^+^ CD11c^+^ macrophage and neutrophil counts in the lung (**b**), and absolute numbers of neutrophils and IgM titers in the BAL (**c**) in hypoxic LPS-challenged mice treated with four daily injections of PBS or CSF-1–Fc. **d**,**e**, Representative lung CD64^hi^SiglecF^−^Ly6C^+^ MDM CD45.2 and CD45.1 expression (**d**) and CD64^hi^SiglecF^−^Ly6C^+^ MDM:blood monocyte chimerism and CD64^hi^SiglecF^−^Ly6C^−^ macrophage:blood monocyte chimerism (**e**) in lung-protected, naive or LPS-challenged mice that are normoxia or hypoxia housed and treated with PBS or CSF-1–Fc. **f**, Differentially expressed genes in PBS- or CSF-1–Fc-treated, LPS-challenged mice in Ly6C^hi^ blood monocytes at day 5 post-LPS challenge. **g**, Overlap between differentially downregulated genes in ARDS blood monocytes and genes upregulated in CSF-1–Fc-treated mice relative to PBS-treated mice. **h**, Comparison of Ly6C^hi^ blood monocyte proteomes from hypoxic, LPS-challenged, CSF-1–Fc-treated mice relative to the PBS-treated counterparts (granule-associated proteins identified). **i**, Representative Lyve1 expression and number of lung CD64^hi^SiglecF^−^Ly6C^−^Lyve1^+^MHC-II^−^ macrophages in naive or LPS-challenged mice housed in normoxia or hypoxia and treated with PBS or CSF-1–Fc for 5 d. **j**, Chimerism of CD64^hi^SiglecF^−^Ly6C^−^Lyve1^+^MHC-II^−^ macrophages to blood monocytes in lung-protected, LPS-challenged chimeras housed in normoxia or hypoxia, treated with PBS or CSF-1–Fc. **k**,**l**, Serum IL-10 (**k**) and representative tiled immunofluorescence of lung sections stained for F4/80, IL-10 and DAPI (**l**), and F4/80, Lyve1 and DAPI in LPS-challenged mice housed in hypoxia for 5 d and treated with CSF-1–Fc or PBS. Scale bar, 200 μm. **m**, Differentially regulated genes in CD64^hi^SiglecF^−^Lyve1^+^MHC-II^−^ macrophages from LPS-challenged, CSF-1–Fc-treated mice relative to CD64^hi^SiglecF^−^MHC-II^−^ macrophages from PBS-treated counterparts, housed in hypoxia for 5 d. **n**–**p**, Total lung CD64^hi^SiglecF^−^ macrophages and CD64^hi^SiglecF^−^Ly6C^−^Lyve1^+^MHC-II^−^ macrophages (**n**), BAL MDMs (**o**) and body-weight change (relative to baseline) (**p**) in LPS-challenged *Ifnar1*^−/−^ mice treated with PBS (*Ifnar1*^−/−^ PBS) or CSF-1–Fc (*Ifnar1*^−/−^ CSF-1) for 5 d. Data represent the mean ± s.e.m. Each datapoint represents an individual mouse. Statistics for **b**,**c**,**k** and **n**–**p** were by unpaired, two-sided Student’s *t*-test, for **i** and **j** by a one-way ANOVA with Tukey’s post-test and for **e** by a two-tailed Mann–Whitney *U*-test following D’Agostino and Pearson’s normality test. For **b** and **c** data are pooled from three independent experiments, and for **f**,**k**,**j** and **n**–**p** data are pooled from two independent experiments. In **g** all genes have a fold-change >1, except H2-DMa, H2-DMb2, IL-17Ra and Nlrp3 where the fold-change is >0.5 and *P* < 0.05.

**Extended Data Fig. 6:**
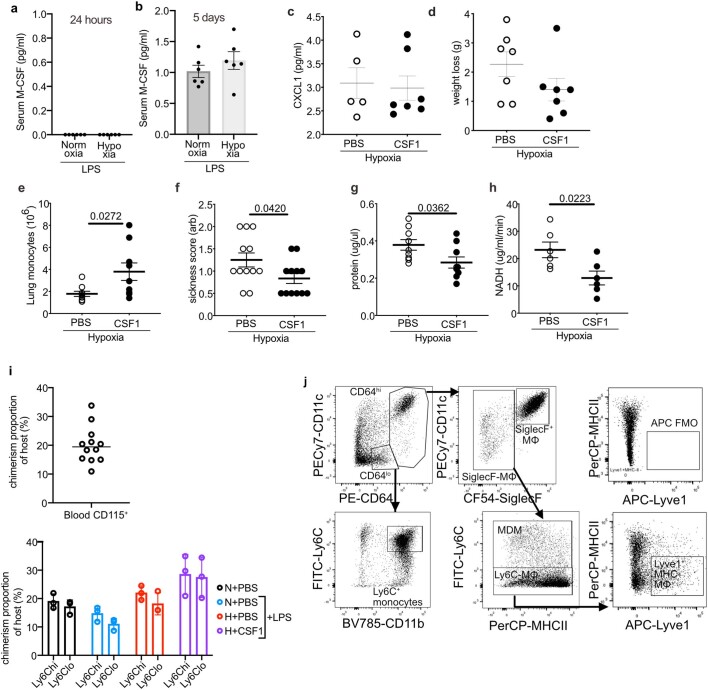
Circulating CSF1 is unchanged in hypoxic ALI and exogenous CSF1 improves injury outcomes altering the CD64^hi^SiglecF^−^ Mϕ phenotype. Serum MCSF (CSF1) from LPS-challenged mice housed in normoxia (N) or hypoxia (H) for 24 hours (**a**) or 5 days (**b**) was measured. (**c**) BAL CXCL1 measured in LPS-challenged mice housed in hypoxia for 5 days and treated with PBS or CSF1-Fc (H CSF1). (**d**) weight loss from baseline in hypoxic LPS-induced ALI treated with PBS (H PBS) or CSF1-Fc (H CSF1). (**e**) Lung monocyte numbers, (**f**) arbitrary sickness scores, (**g**) BAL protein and (**h**) LDH activity (as measured by NADH) measured at 48 hours in mice with virally-induced ALI housed in hypoxia and treated with PBS or CSF1-Fc (H CSF1). (**i**) Baseline blood chimerism (proportion of donor cells relative to host) of circulating monocytes in lung-protected chimeras prior to ALI induction and chimerism of monocytes based on Ly6C expression post-LPS. **(j**) Lung cDC1 (gated on Alive CD45^+^Lin-CD64^−^CD11c^+^Cd103^+^) chimerism and counts and (**k**) cDC2 (gated on Alive CD45^+^Lin-CD64^−^CD11c^+^Cd103^−^ CD11b^+^) chimerism and counts. Chimerism relative to blood monocyte chimerism. (**l**) *Il10* expression was measured by NanoString platform analysis in MHC-lung macrophages from LPS-challenged mice housed in hypoxia for 5 days and treated with PBS and compared to Lyve1+MHCII- of LPS-challenged mice, housed in hypoxia and treated with CSF1-Fc. (**m**) Representative dot plots of lung digests showing gating strategy for identification of the different monocyte and macrophage populations in the lung gated on Live Singles CD45^+^Ly6G^−^ cells, and including Lyve1^+^MHC-CD64^hi^SiglecF^−^Mϕ, and associated APC FMO control including Lyve1^+^MHC-CD64^hi^SiglecF^−^Mϕ, and associated APC FMO control. Ifnar^−/−^ (KO) mice were nebulised with LPS and treated with PBS (KO PBS) or CSF1-Fc (KO CSF1). Mice were sacrificed on day 5 and (**n**) blood monocyte and (**o**) lung Ly6C^+^ monocytes were quantified by flow cytometry. **c**, **d** Mean±SD, **e**-**h** mean±SEM. **c**-**h, j, k** 2 pooled experiments, **l** representative of 3 experiments, **n**, **o** representative of 2 experiments. Statistical testing **e**-**h, n, o** unpaired two-sided t-test, **k**, one-way ANOVA with Tukey’s multiple comparisons test.

Finally, we tested whether increasing the number of monocytes during hypoxia facilitated inflammation resolution. LPS-challenged hypoxic mice with absent (24 h) or low (5 d) baseline levels of macrophage-colony-stimulating factor (M-CSF) (Extended Data Fig. 6a,b) were treated with four daily injections of CSF-1–Fc fusion protein^34^ or phosphate-buffered saline (PBS). CSF-1–Fc markedly increased the number of CD115^+^CD11b^+^ monocytes and moderately increased the number of Ly6G^+^CD11b^+^ neutrophils in the blood at day 5 compared with PBS (Fig. 7a). CSF-1–Fc-treated, hypoxic, LPS-challenged mice also had increased numbers of CD64^lo^CD11b^+^Ly6C^+^ monocytes and CD64^hi^SiglecF^–^ macrophages in the lung compared with mice receiving PBS (Fig. 7b). The number of CD64^hi^SiglecF^+^CD11c^+^ macrophages was not affected (Fig. 7b). Importantly, the absolute number of Ly6G^+^CD11b^+^ neutrophils in the lung tissue (Fig. 7b) and BAL (Fig. 7c) was reduced in CSF-1–Fc-treated, hypoxic, LPS-challenged mice at day 5, despite equivalent levels of CXCL1 in the BAL between CSF-1–Fc-treated and PBS-treated mice (Extended Data Fig. 6c). Hypoxic LPS-challenged mice treated with CSF-1–Fc had reduced weight loss (Extended Data Fig. 6d) and reduced immunoglobulin (Ig)M levels in the BAL fluid at day 5 (Fig. 7c) compared with PBS counterparts, suggesting reduced alveolar inflammation and vascular leak. To test these observations in a model of virally-induced epithelial injury, C57/BL6J mice were inoculated with influenza A virus (PR8) and placed in hypoxia immediately after. CSF-1–Fc or PBS was administered 12 h and 36 h post-PR8 challenge. Hypoxic PR8-infected mice treated with CSF-1–Fc had increased numbers of CD64^lo^CD11b^+^Ly6C^hi^ monocytes in the lung (Extended Data Fig. 6e) and improved physiological outcomes (Extended Data Fig. 6f) compared with mice receiving PBS. This was associated with a significant reduction in BAL protein levels, a marker of lung injury (Extended Data Fig. 6g) and lactate dehydrogenase activity, as an indicator of cellular damage (Extended Data Fig. 6h).

To dissect the mechanism by which CSF-1–Fc accelerated inflammation resolution, we tracked the ontogeny of CD64^hi^SiglecF^–^ macrophages in CD45.1^+^CD45.2^+^ C57BL6 mice reconstituted with CD45.2^+^ BM cells after lung-protected, single-dose irradiation. Then 8 weeks after BM reconstitution (Extended Data Fig. 6i), the mice were challenged with LPS, housed in normoxia or hypoxia for 5 d and treated with PBS or CSF-1–Fc daily. In keeping with increased recruitment from the blood, the proportion of CD64^hi^SiglecF^–^Ly6C^+^ MDMs derived from donor CD45.2^+^ BM cells in LPS-treated mice paralleled that seen in the blood (Fig. 7d,e). In the absence of LPS challenge, the proportion of CD45.2^+^CD64^hi^SiglecF^−^Ly6C^−^ macrophages relative to the CD45.2^+^CD115^+^CD11b^+^ blood monocyte pool was ~20%, indicating that maintenance of this subset was dependent on blood monocytes (Fig. 7e)^35^. In LPS-treated mice housed in normoxia, the proportion of CD45.2^+^CD64^hi^SiglecF^−^Ly6C^−^ macrophages relative to CD45.2^+^CD115^+^CD11b^+^ blood monocytes was ~80% (Fig. 7e), suggesting that the expansion of this population during inflammation was predominantly through recruitment of blood monocytes. The chimerism of this population was ~60% in hypoxic, LPS-challenged, PBS-treated mice and ~80% in hypoxic, LPS-challenged mice treated with CSF-1–Fc (Fig. 7e), indicating that CSF-1–Fc replenished the number of CD64^hi^SiglecF^–^ lung macrophages in LPS-treated hypoxic mice predominantly through increased recruitment of circulating CD115^+^CD11b^+^ monocytes.

In LPS-treated hypoxic mice, CSF-1–Fc treatment also normalized the hypoxic suppression of the type I IFN-associated gene *Ccr5*, without enhancing expression of *Il1b* (Fig. 7f). CSF-1–Fc significantly induced the expression of a number of genes that were suppressed in the ARDS patient samples, such as *Itga5, Cd99*, *Sell* and *Anxa*^36^ (Fig. 7g and Supplementary Table 1). A proteomic survey showed reduced abundance of secretory-granule proteins in circulating Ly6C^hi^ monocytes of LPS-challenged, hypoxic, CSF-1-treated compared with PBS-treated counterparts (Fig. 7h), suggesting that CSF-1 altered their phenotype toward a less inflammatory profile. Increased numbers of lung MHC-II^–^Lyve-1^+^ macrophages, a subset of repair-associated macrophages reported in bleomycin-induced lung injury^11^, were observed in CSF-1–Fc-treated, hypoxic, LPS-challenged mice compared with their PBS counterparts (Fig. 7i and Extended Data Fig. 6j), with increased chimerism noted in the CSF-1–Fc-treated mice in the lung-protected chimera model (Fig. 7j)

**Extended Data Fig. 7:**
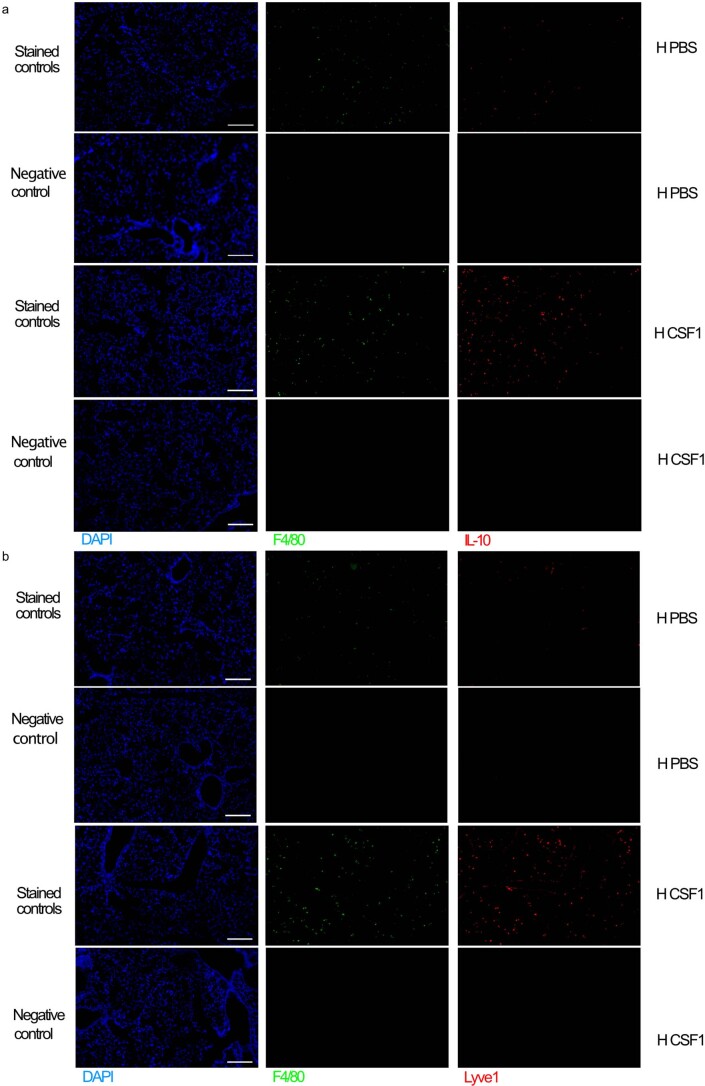
Lung immunohistochemistry tiled separate-channel images with negative controls. (**a**, **b**) Representative images of separate channels for stained tissue and negative controls (no primary antibody) for the indicated antibodies in lung sections from mice housed in hypoxia post-LPS and treated with either PBS or CSF1 as indicated (n = 3/ group). Scale bar represents 200μm.

**Extended Data Fig. 8:**
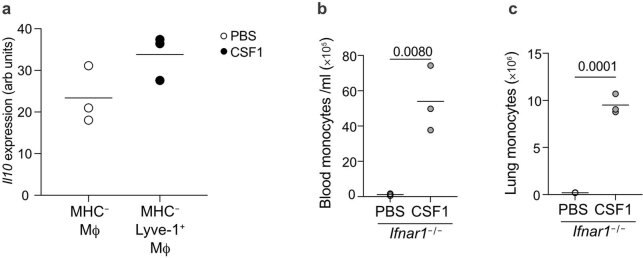
Treatment with CSF-1-Fc elevates *II10* expression in Lyve1^+^MHCII^−^ macrophages and increases blood and lung monocyte numbers in *Ifnar1*^*–/–*^ mice. (**a**) *II10* expression was measured by NanoString platform analysis in MHC^−^ lung macrophages from LPS-challenged mice housed in hypoxia for 5 days and treated with PBS and compared to Lyve1^+^MHCII^−^ of LPS-challenged micehoused in hypoxia and treated with CSF-1-Fc. (**b, c**) *Ifnar1*^−/−^ mice were nebulized with LPS and treated with PBS or CSF-1-Fc. Mice were sacrificed on day (**b**) blood monocyte and (**c**) lung Ly6C^+^ monocytes were quantified by flow cytometry. (**a–c**) representative of 2 experiments. Statistical testing (**a–c**) unpaired, two-sided Student’s *t*-test.

To explore how CSF-1–Fc-mediated expansion of CD64^hi^SiglecF^−^ macrophages facilitated neutrophil clearance, we investigated the expression of known mediators of efferocytosis in the blood and lung^37^. IL-10 was elevated in the serum of CSF-1–Fc-treated, hypoxic, LPS-challenged mice compared with the PBS-treated counterparts (Fig. 7k). Immunofluorescence staining showed increased numbers of IL-10-expressing interstitial F4/80^+^ cells (Fig. 7l and Extended Data Fig. 7a), and F4/80^+^Lyve1^+^ macrophages (Fig. 7l and Extended Data Fig. 7b) in the lung of hypoxic, LPS-challenged, CSF-1–Fc-treated mice compared with their PBS-treated counterparts. Sorted CD64^hi^SiglecF^−^MHC-II^−^Lyve1^+^ macrophages from these CSF-1–Fc-treated mice expressed the *Il-10* transcript (Extended Data Fig. 8a). Transcriptional profiling indicated that CD64^hi^SiglecF^−^MHC-II^−^Lyve1^+^ macrophages from CSF-1–Fc-treated, LPS-challenged, hypoxic mice had lower expression of archetypal inflammatory genes (*Il1b*, *Il6*, *Tnf* and *Il18* and *S100a11*) and genes associated with lung fibrosis (*Mmp8*, *Mmp12*, *Col14a1*, *Fpr1*, *Pdgfa* and *Pdgfb)* relative to CD64^hi^SiglecF^−^MHC-II^−^ from PBS-treated counterparts. Furthermore, genes reported to be enriched in severe SARS-CoV2 infection in BAL monocytes or macrophages, such as *CD14*, *CCL3* and *S100A8* (ref. ^38^), were suppressed in the CD64^hi^SiglecF^−^MHC-II^−^Lyve1^+^ macrophages from the CSF-1–Fc-treated hypoxic mice compared to CD64^hi^SiglecF^−^MHC-II^−^ macrophages from the PBS-treated counterparts (Fig. 7m), indicating that CSF-1 expanded the number of IL-10-producing macrophages and enabled resolution of hypoxia-driven inflammation.

Finally, we asked whether CSF-1 was sufficient to overcome the loss of CD64^hi^SiglecF^–^Ly6C^+^ MDMs and CD64^hi^SiglecF^−^ macrophages observed in the absence of type I IFN signaling. Normoxic LPS-challenged *Ifnar1* ^−*/*−^ mice treated with CSF-1–Fc (four daily injections) had increased numbers of CD115^+^CD11b^+^ monocytes in the blood, Ly6C^hi^ monocytes (Extended Data Fig. 8b,c) and CD64^hi^SiglecF^−^ macrophages in the lung (Fig. 7n) compared with those receiving PBS. In addition, these mice had increased lung CD64^hi^SiglecF^−^Lyve1^+^ macrophages (Fig. 7n) and BAL-recovered CD64^hi^SiglecF^*–*^ MDM numbers (Fig. 7o), with enhanced return to baseline body weight at day 5 post-LPS challenge (Fig. 7p) compared with LPS-challenged, PBS-treated *Ifnar1*^−/−^ mice.

## Discussion

In the present study, we showed that monocyte recruitment and conversion into lung macrophages are required to drive inflammation resolution in hypoxic ALI. Hypoxemic mice with ALI demonstrated an increase in erythropoiesis, with an associated reduction in monopoiesis, monocytopenia and failure to expand the MDM and non-AM CD64^hi^SiglecF^−^macrophage compartment in the lung. In the context of prioritizing the preservation of tissue oxygen delivery, increased erythropoiesis makes physiological sense, such as in adaptation to altitude, where monocytopenia had been reported as early as 1969 (ref. ^39^). However, when engagement of an effective innate immune response is also required, our data demonstrated that hypoxia-induced immune changes observed in early disease have long-term consequences for inflammation resolution, such as persistence of neutrophilic inflammation, a well-known poor prognostic feature of ARDS^3^.

Monocytes are professional phagocytes and key mediators of the restoration of homeostasis. There is increasing appreciation that the phenotype of circulating monocytes can be predetermined by systemic cues affecting their BM progenitors^40^. The presence of a specific phenotypic profile in ARDS patients’ circulating monocytes, irrespective of the sampling timepoint, would be in keeping with alterations within their progenitors. It will be important, in future work, to explore whether the absence of systemic IFN-α, and suppressed IFNAR expression on BM LSK and circulating monocytes, as observed in our mouse model of hypoxic ALI and in hypoxemic patients with severe SARS-Cov2 infection^41,42^, may be sufficient to drive the phenotypic and functional changes observed in the circulating monocytes of a hypoxic ARDS cohort.

Expanded numbers of airway monocytes and macrophages in the BAL of patients with severe SARS-Cov2 infection have been reported^38^, although it is unclear whether this expansion is promoting or limiting disease pathogenesis. An important limitation of our present study is the inability to sample the lung macrophage compartment in patients with ARDS. Transcriptomic survey of the airway monocytes and macrophages in patients with severe SARS-Cov2 infection identified an enrichment of markers of immaturity, inflammatory proteins and cytokines^38^. We show that treatment with CSF-1 increased the number of CD64^hi^SiglecF^−^ macrophages and monocyte differentiation toward CD64^hi^SiglecF^−^MHC^−^Lyve1^+^ macrophages, a cell type that has tissue-repair roles in various disease contexts^43^, including in the lung^11^. In addition, CSF-1–Fc suppressed the expression of several genes reported to be enriched in the BAL of patients with severe SARS-Cov2 infection^38^. These findings, compounded by the effect of CSF-1–Fc on accelerating inflammation resolution and reducing lung injury, underscored the therapeutic potential of CSF-1 in ARDS. The use of other growth factors has been trialed in ARDS without success, with systemic-^44^ or lung-delivered^45^ granulocyte–macrophage colony-stimulating factor (GM-CSF) failing to improve ARDS mortality. Although GM-CSF plays a key role in AM homeostasis, its pleotropic nature means that it can also act as a neutrophil chemoattractant and growth factor^46^, with neutrophils pathogenic in the context of ARDS and hypoxia promoting neutrophil survival and proinflammatory function^32,33^. On the other hand, monocytes and MDMs can directly inhibit neutrophil-mediated damage to the host^47^. In our system, CSF-1–Fc treatment expanded the CD64^hi^SiglecF^−^ macrophage compartment and led to an increase in lung IL-10^+^ macrophages, with a concomitant increase in systemic IL-10, and reduced numbers of neutrophils in the airspace and the lung. Macrophages are known to release IL-10 on efferocytosis, which drives the resolution of inflammation^37^. Furthermore, IL-10-producing lung IMs are key regulators of both allergic^8,9^ and endotoxin-mediated lung injury^10^. These findings strengthen the case of the therapeutic potential of CSF-1 in human ARDS.

## Methods

### Resources availability

#### Lead contact

Further information and requests for resources and reagents should be directed to and will be fulfilled by the lead contact, A.M. (Ananda.Mirchandani@ed.ac.uk).

#### Human healthy control blood donors

Patients with ARDS were recruited and informed consent obtained directly or by proxy under the ‘META-CYTE’ study (17/SS/0136/AM01) and ‘ARDS-NEUT’ study (20/SS/0002), as approved by the Scotland A Research Ethics Committee. Samples were also obtained under the ‘Effects of Critical Illness on the Innate Immune System’ study as approved by Health Research Authority (REC no. 18/NE/0036).

All healthy participants gave written informed consent in accordance with the Declaration of Helsinki principles, with ACCORD Medical Ethics Research Committee approval for the study of healthy human volunteers through the University of Edinburgh Centre for Inflammation Research blood resource (15-HV-013).

Up to 20–40 ml of whole blood was collected into citrate tubes and up to 10 million cells were stained for flow cytometry assessment and sorting. Briefly, the whole blood was treated with red cell lysis buffer (Invitrogen) and cells counted before staining for flow cytometry. Cells were incubated with anti-CD16/32 Fc block (2:50) for 30 min, followed by staining for 30 min with antibodies (Table 2) followed by a wash with FACS buffer (PBS + 2% fetal calf serum (FCS)). DAPI (1:1,000) was added before flow cytometry to determine live cells. Monocytes were identified as Singles Dapi^−^CD45^+^ nongranulocyte Lin(CD3/CD56/CD19^±^CD66b)^−^ HLADR^+^ CD14^+^ and/or CD16^+^ cells.

**Table 2:** List of antibodies

Antibody	Clone no.	Catalog no.	Lot no.	Fluorophore	Source	Dilution
CD16	eBioCD16	1-9161-71	4304474	FITC	eBioscience	1:20
CD3	OKT3	317308	B256076	PE	BioLegend	1:80
CD56	HCD56	318306	B252053	PE	BioLegend	1:80
CD19	HIB19	302254	B227178	PE	BioLegend	1:200
CCR2	K036C2	357212	B260108	PE/Cy7	BioLegend	1:80
ICAM	HCD54	322718	B193832	AF647	BioLegend	1:80
CD45	2D1	368514	B248834	AF700	BioLegend	1:20
CD14	M5E2	301820	B274258	APC/Cy7	BioLegend	1:20
HLA-DR	L243	307624	B278326	Pacific Blue	BioLegend	1:20
CD66b	G10F5	305106	B278603	PE	BioLegend	1:20
SiglecF	E50-2440	552126	7058859	PE	BD Biosciences	1:200
CD11b	M1/70	101256	B238075	PE Dazzle	BD Biosciences	1:400
CD11b	M1/70	101243	B253527	BV785	Biolegend	1:200
MHC-II	M5.114.15.2	107624	B267551	PerCP	eBioscience	1:200
Epcam	G8.8	118230	B251914	APCCy7 Fire	BioLegend	1:200
CD3	17A2	100244	B198733	BIOTIN	BioLegend	1:200
CD3	17A2	100213	B261416	Pacific Blue	BioLegend	1:200
CD3	17A2	100229	B282101	BV650	BioLegend	1:200
CD3	17A2	100206	B210714	PE	BioLegend	1:200
CD19	6D5	115541	B242632	BV650	BioLegend	1:200
CD19	6D5	115504	B244881	Biotin	BioLegend	1:200
CD19	6D5	115526	B265435	Pacific Blue	BioLegend	1:200
CD19	6D5	115508	B223615	PE	BioLegend	1:200
CD103	2E7	121433		BV605	BioLegend	1:400
Ly6G	1A8	127604	B218526	Biotin	BioLegend	1:200
Ly6G	1A8	127608	B221647	PE	BioLegend	1:200
Ly6G	1A8	127628	B280589	BV421	BioLegend	1:200
Ly6G	1A8	135512	B213676	AF488	BioLegend	1:200
Lyve-1	ALY7	50-0443-82	2205461	eFluor 660	eBioscience	1:200
CD115	AFS98	135510	B211309	APC	BioLegend	1:200
CD115	AFS98	128006	B217035	FITC	BioLegend	1:200
Ly6C	HK1.4	128032	B232012	BV421	BioLegend	1:200
Pan-CD45	30-F11	103128	B274307	AF700	BioLegend	1:200
CD11c	N418	117318	B222652	PE/Cy7	BioLegend	1:200
CD11c	N418	117352	B218048	APC/Fire750	Biolegend	1:200
CD64	X54-5/7.1	139304	B191540	PE	BioLegend	1:200
CD64	X54-5/7.1	139306	B207411	APC	BioLegend	1:200
CD4	H129.19	553649		Biotin	BD Biosciences	1:1,600
CD5	53-7.3	553019		Biotin	BD Biosciences	1:800
CD5	53-7.3	100603	B254317	Biotin	BioLegend	1:200
CD8a	53 -6.7	553029		Biotin	BD Biosciences	1:800
CD11b	M1/70	101256	B238075	Biotin	BD Biosciences	1:200
CD45R/B220	RA3-6B2	553086		Biotin	BD Biosciences	1:200
Ter119	TER-119	116204	B295203	Biotin	BioLegend	1:200
Ter119	TER-119	553672		Biotin	BD Biosciences	1:50
Gr-1/Ly-6G/C	RB6-8C5	553125		Biotin	BD Biosciences	1:100
CD117/cKit	2B8	105811	B249345	APC	BioLegend	1:200
Sca-1/Ly-	E13-161.7	122506		FITC	BioLegend	1:200
CD48	HM48-1	103406		PE	BioLegend	1:500
CD150	12F12.2	115914		PECy7	BioLegend	1:200
CD71	RI7217	113807		PE	BioLegend	1:500
Fc Block CD16/32	93	101320	B295040		BioLegend	1:100
Streptavidin	–	405232	B251688	BV650	BioLegend	1:1,000
Streptavidin	–			Pacific Blue	BD biosciences	
LIVE/DEAD Fixable Aqua	–	L34957	2068285	UV650	Life Technologies or BioLegend	1:50–1:100
CD45.2	104	109822	B252126	AF700	BioLegend	1:200
CD45.1	A20	110741	B253101	BV510	BioLlegend	1:200
Sca.1	D7	108129	B262926	BV510	BioLegend	1:200
CD150	TC15-12F12.2	115903		PE	BioLegend	1:200
CD105	MJ7/18	120412	B245562	PacBlue	BioLegend	1:200
CD41	MWReg30	133927	B268849	APCCy7	BioLegend	1:200
IFNAR	MAR1-5A3	127325	B286788	PECy7	BioLegend	1:200
CD5	53-7.3	100603	B254317	Biotin	BioLegend	1:200
Ly6G	1A8	127604	B218529	Biotin	Biolegend	1:200
B220	RA3-6B2	103204	B288658	Biotin	BioLegend	1:200
CD11b	M1/70	562287		CF594	BioLegend	1:200
F480	CI:A3-1	ab6640		Purified	Abcam	1:100
IL-10	JES5-2A5	ab189392		Purified	Abcam	1:100
LYVE-1	Polyclonal	103-PA50AG		Purified	ReliaTech GmbH	1:200
HIF-1α	Polyclonal	NB100-479		Purified	Novus Biotech	1:00
CD11b	M1/70	101243	B287244	BV785	BioLegend	

Samples obtained from April 2020 were fixed before acquisition given the potential for SARS-Cov2 dissemination. Briefly, 1 μl of Zombie Aqua fixable viability dye (stock 1:20 dilution) was added to 100 μl of whole blood for 15 min at room temperature in the dark. Then, 2 μl of Fc Block was added for a further 30 min, on ice. Samples were then stained as above and fixed/lysed using BD FACS Lyse for 10 min at room temperature. The sample was then resuspended in 300 μl of FACS buffer and 50 μl of Countbright beads added (Thermo Fisher Scientific) before acquisition.

#### Mice

Male C57/BL6J mice aged 8–15 weeks were purchased from Envigo or Charles River. *Ifnar1*^−*/*−^ (*ifnar* ^*tm/agt*^) mice were obtained from J.S. who purchased them originally from the Jackson Laboratory. Animal experiments were conducted in accordance with the UK Home Office Animals (Scientific Procedures) Act of 1986 with local ethical approval.

#### Mouse LPS ALI model

Mice were treated with nebulized LPS (3 mg) and then housed in normoxia or hypoxia (10% O_2_) immediately thereafter for up to 5 d. Mice were treated daily (days 1–4 post-LPS), by subcutaneous injection, with PBS or 0.75 mg kg^−1^ of porcine CSF-1 fused to the Fc region of porcine IgG1a (generated by David Hume), prior to cull on day 5.

#### D39 *S. pneumoniae* infection

Mice were anesthetized and 10^7^ colony-forming units (c.f.u.) (or vehicle) was delivered in 50 μl of PBS via intratracheal intubation. After reversal of the anesthetic and a period of recovery, the mice remained in normoxia or were placed in hypoxia.

#### Influenza A (PR8) virally induced ALI model

Mice were lightly anesthetized using isoflurane and 20 plaque-forming units (p.f.u.) of PR8 influenza A virus in Dulbecco’s modified Eagle’s medium (DMEM) was inoculated intranasally. After 1 h of recovery time, mice were placed in hypoxia for 48 h. Subcutaneous PBS or CSF-1–Fc injections (as above) at 12 h and 36 h were administered. Sickness scores were determined using methods described previously^32^.

#### Lung and alveolar cell sampling

Mice were culled with an overdose of intraperitoneal anesthetic (Euthetal) followed by blood collection from the inferior vena cava. Alveolar leukocytes were collected by BAL, then mice were perfused lightly with PBS through the heart, before harvesting the lung tissue. On occasion, the lower limbs were harvested for BM leukocyte assessment (see below).

Tissue leukocytes were extracted from surgically dissociated lung tissue by enzymatic digestion with 2 ml of enzyme mix (RPMI with 0.625 mg ml^−1^ of Collagenase D (Roche), 0.85 mg ml^−1^ of Collagenase V (Sigma-Aldrich), 1 mg ml^−1^ of dispase (Gibco, Invitrogen) and 30 U ml^−1^ of DNase (Roche Diagnostics GmbH)) for 45 min at 37 °C in a shaking incubator. The digest material was passed through a 100-μm cell strainer with the addition of FACS buffer (PBS with 0.5% BSA/2% FCS and 0.02 mM EDTA). Cell pellets were treated with red cell lysis buffer (Sigma-Aldrich) and washed in FACS buffer. The resulting cell suspension was subsequently passed through a 40-μm strainer before cell counting using a Casey TT counter (Roche). Single-cell suspensions (5 million cells per sample) were then stained for flow cytometry. BAL samples were counted before staining for flow cytometry.

#### Blood and BM sampling

Mouse blood and BM were treated with RBC lysis buffer (BioLegend) before counting and staining for flow cytometry (Table 2).

Hematopoietic cell assessment was performed using both hind legs, which were crushed using a pestle and mortar until a homogeneous cell suspension was achieved, or flushed though using a 32G needle. Cells were collected in cold FACS buffer and filtered through a 70-μm nylon strainer (BD Falcon, catalog no. 352340). Cells were treated with RBC lysis buffer (BioLegend) before staining.

#### Tissue-protected chimeras

C57BL/6J CD45.1^+^CD45.2^+^ mice aged 6–8 weeks were anesthetized and irradiated with a single dose of 9.5-Gy γ-irradiation, with all but the hind legs and lower abdomen protected by a 5cm lead shield. The next day, the mice received 2 × 10^6^–5 × 10^6^ BM cells from CD45.2^+^ C57BL/6J by intravenous injection. The chimerism of blood monocytes (proportion of donor cells) was determined by flow cytometry in each individual mouse at day 5 and the chimerism in the lung macrophage populations (as described in the figures) was divided by this reference value, thereby determining the proportion of the cells that were of blood ontogeny.

#### Flow cytometry

Mouse cells were treated with α-CD16/32 Fc block (eBioscience) (1:100) before staining with antibodies (Table 2). Relevant fluorescence − 1 samples were used as controls. Zombie Aqua fixable viability dye (BioLegend) was used before Fc Block to exclude dead cells from digest samples or DAPI for single-cell suspensions.

Cells were acquired on the LSRFortessa (Becton Dickinson) or sorted on an Aria II or Fusion machine (Becton Dickinson). Compensation was performed using BD FACSDiva software and data analyzed in FlowJo v.10 or FCS Express 7 for *t*-distributed stochastic neighbor embedding analysis.

#### Gating strategies

- Human monocytes: Singles Dapi^−^CD45^+^non-granulocyte Lin(CD3/CD56/CD19/CD66b)^−^ HLADR^+^ CD14^+^ and/or CD16^+^ cells.
- Mouse blood monocytes: Singles Dapi^−^CD45^+^ Lin(CD3/CD19/ Ly6G)^−^CD115^+^CD11b^+^Ly6C^hi^, Ly6C^int^ or Ly6C^−^.
- Mouse blood neutrophils: Singles, Dapi^−^CD45^+^Ly6G^+^CD11b^+^Ly6C^int^.
- Mouse lung/BAL alveolar macrophages: Singles, Zombie Aqua^−^CD45^+^Lin (CD3/CD19/Ly6G)^−^CD64^hi^SiglecF^+^CD11c^+^.
- Mouse lung interstitial /BAL inflammatory macrophages: Singles, Zombie Aqua^−^CD45^+^Lin (CD3/CD19/Ly6G)^−^CD64^hi^SiglecF^−^CD11c^+/−^ then Ly6C^+/−^MHC-II^+/−^.
- Lung classical monocytes: Singles, Zombie Aqua^−^SinglesCD45^+^Lin (CD3/CD19/Ly6G)^−^CD64^lo^ CD11b^+^Ly6C^+^.
- Lung/BAL neutrophils: Singles, Aqua or Dapi^−^, CD45^+^CD11b^+^Ly6G^+^.
- Lung cDC1 subset: Zombie Aqua-Singles CD45^+^CD11c^hi^,CD103^+^, CD64^−^MHC-II^+^.
- BM HSPC SLAM analysis Alive: Singles LK (Lin-cKit^+^) and LSK (Lin-cKit^+^Sca-1^+^) cells. LSK cells were further sub-gated on hematopoietic stem cells (HSCs: LSK CD48^−^CD150^+^), multipotent progenitors (MPPs: LSK CD48^−^CD150^−^), HPC-1 (LSK CD48^+^CD150^−^) and HPC-2 (LSK CD48^+^CD150^+^).
- BM erythroid progenitors based on Pronk analysis^26^: Singles, Dapi or Aqua^−^, Lin^−^, CD11b^−^, cKit^+^, Sca1^−^, CD32/16^−^, CD41^−^, CD105^+^ or CD150^+^ (pre-Meg-E CD150^+^CD105^−^, pre-CFU-E CD150^+^CD105^+^, CFU-E CD150^−^CD105^+^).

Further gating strategy information can be made available on request.

#### BAL/serum cytokine/chemokine quantification

BAL and serum supernatants were collected and stored at −80 °C until use. Cytokine and chemokine levels were measured using an MSD V-plex plate per the manufacturer’s instructions.

#### Lung injury measurements

IgM BAL levels were measured using the Ab133047 Abcam kit as per the manufacturer’s instructions.

BAL lactate dehydrogenase activity (measured as colorimetric reduction of NAD to NADH) was performed using Ab102526 (Abcam) as per the manufacturer’s instructions.

BAL total protein was measured using Pierce BCA Assay (Thermo Fisher Scientific) as per the manufacturer’s instructions.

#### In vitro BM culture

Naive WT C57BL/6 BM was obtained by flushing the femoral and tibial bones and RBCs were lysed. Cells were cultured in hypoxia (*F*iO_2_ 1%) or normoxia (*F*iO_2_ 21%) with conditioned DMEM for 1 h before the addition of IFN-β 10 ng ml^−1^ (RnD 8234-MB-010) for a further 3 h. Cell pellets were collected and QIAGEN RLT buffer added (containing 10 μl ml^−1^ of 2-mercaptoethanol). Pellets were snap-frozen and stored at −80 °C for RNA extraction.

#### RNA isolation and relative quantification

RNA was isolated from BM cells using the genomic DNA eliminator solution for purification of total RNA (RNeasy Plus Mini Kit, QIAGEN). Complementary DNA was synthesized using AMV reverse transcriptase with random primers (Promega). TaqMan gene expression assays (Applied Biosystems, Thermo Fisher Scientific) and PrimeTime qPCR Probe Assays (IDT) were used for relative quantification of cDNA using SDS 2.4 (Thermo Fisher Scientific) and normalized to ACTB expression.

#### Immunohistochemistry

Murine paraffin-embedded blocks were prepared from lungs fixed via the trachea with 10% buffered formalin. The lung sections were stained with anti-IL-10 (catalog no. ab189392, Abcam), anti-F4/80 (catalog no. ab6640, Abcam) or isotype control after deparaffinization and antigen retrieval. Antigen retrieval was performed by microwave heating in citric acid-based, antigen-unmasking solution (Vector, catalog no. H-3300-250). The following were used: tyramide signal amplification (TSA) plus system amplification (catalog no. NEL744B001KT, Perkin Elmer) and autofluorescence quenching with TrueView (Vector, catalog no. SP-8400). The nuclei were stained with DAPI (catalog no. 422801, Sigma-Aldrich). Images were obtained using EVOS FL Auto 2 (Invitrogen). All image acquisition and processing steps were performed using the same settings for both sample groups.

The lung sections were stained with anti-mouse LYVE-1 (catalog no. 103-PA50AG, ReliaTech GmbH) and anti-mouse F4/80 (catalog no. ab6640, Abcam) overnight at 4 °C after deparaffinization and antigen retrieval. Antigen retrieval was performed by microwave heating in citric acid-based, antigen-unmasking solution (Vector, catalog no. H-3300-250). The following were used: TSA plus system amplification (catalog no. NEL744B001KT, Perkin Elmer) and autofluorescence quenching with TrueView (Vector, catalog no. SP-8400) according to the manufacturer’s instructions. The nuclei were stained with DAPI (catalog no. 422801, Sigma-Aldrich). Images were acquired using a EVOS FL Auto 2 (Invitrogen).

All image acquisition and processing steps were performed using the same settings for both sample groups.

#### The nCounter NanoString platform analysis

For human monocytes, 5,000 HLADR^++^ cells were sorted using the aforementioned human monocyte gating strategy directly into 2 μl of RLT buffer using a BD Fusion Sorter (patients 4–8 were sampled). 5,000 mouse classical monocytes were sorted from mice treated with LPS and housed in normoxia, hypoxia and hypoxia + CSF-1 gating on single DAPI^−^CD45^+^Lin^−^CD115^+^Ly6C^hi^ cells into 2 μl of RLT. Cell pellets were vortexed and centrifuged before immediate freezing until ready for processing. NanoString gene expression plates of human and mouse myeloid inflammation were run as per the manufacturer’s instructions at the University of Edinburgh HTPU Centre within the MRC Institute of Genetics and Molecular Medicine/Cancer Research UK Edinburgh Centre.

#### Proteomic analysis

Sorted classical monocytes were processed for proteomics using the ‘in-cell digest’, as described by Kelly et al.^48^, resuspended in digestion buffer (0.1 M triethylammonium bicarbonate + 1 mM MgCl_2_) and digested with benzonase (>99%, Millipore) for 30 min at 37 °C, followed by trypsin (Thermo Fisher Scientific, 1:50 w:w protein) overnight at 37 °C. A second aliquot of trypsin (1:50) was subsequently added and incubated at 37 °C for 4 h. A minimum of 25 ng of trypsin was added. Digests were acidified and desalted using StageTips^49^ and subjected to either tip-based fractionation or direct analysis by liquid chromatography–tandem mass spectroscopy (LC–MS/MS).

After digestion, and to generate the reference spectral library, peptides were subjected to reverse-phase, high pH, tip fractionation following the general guidelines described by Rappsilber et al.^49^. In brief, tips for fractionation were made using three SDB-XC disks (Merck) per tip. The tip was cleaned and conditioned using, sequentially, methanol, 80% acetonitrile (MeCN) (Thermo Fisher Scientific) in 0.1% NH_4_OH (v:v), and 0.1% NH_4_OH (52 mM) (v:v). Peptides, also resuspended in 0.1% NH_4_OH, pH 10, were spun through the SDB-XC disks and the flow-through was collected, acidified and concentrated on C-18 StageTips before being subjected to MS analysis. Fractionation was then achieved by sequential elution with 7%, 14%, 21%, 28%, 35%, 55% and 80% MeCN in 0.1% NH_4_OH. Fractions were then dried at ambient temperature (Concentrator 5301, Eppendorf) and prepared for MS analysis by resuspension in 6 μl of 0.1% trifluoroacetic acid (TFA).

Data-dependent acquisition LC–MS analyses were performed on an Orbitrap Fusion Lumos Tribrid Mass Spectrometer (Thermo Fisher Scientific) coupled, on-line, to an Ultimate 3000 HPLC (Dionex, Thermo Fisher Scientific). Peptides were separated on a 50-cm (2-µm particle size) EASY-Spray column (Thermo Fisher Scientific), which was assembled on an EASY-Spray source (Thermo Fisher Scientific) and operated constantly at 50 °C. Mobile phase A consisted of 0.1% formic acid in LC–MS-grade water and mobile phase B consisted of 80% acetonitrile and 0.1% formic acid. Peptides were loaded on to the column at a flow rate of 0.3 μl min^−1^ and eluted at a flow rate of 0.25 μl min^−1^ according to the following gradient: 2–40% mobile phase B in 120 min and then to 95% in 11 min. Mobile phase B was retained at 95% for 5 min and returned back to 2% a minute after until the end of the run (160 min in total).

The spray voltage was set at 2.2 kV and the ion capillary temperature at 280 °C. Survey scans were recorded at 60,000 resolution (scan range 400–1,600 *m*/*z*) with an ion target of 1.0 × 10^6^ and injection time of 50 ms. MS2 was performed in the orbitrap (resolution at 15,000), with an ion target of 5.0 × 10^4^ and higher-energy C-trap dissociation (HCD) fragmentation^50^ with a normalized collision energy of 27. The isolation window in the quadrupole was 1.4 Thomson. Only ions with a charge between 2 and 6 were selected for MS2. Dynamic exclusion was set at 60 s. The cycle time was set at 3 s.

Samples subjected to data-independent acquisition (DIA) were prepared for MS analysis by resuspension in 0.1% TFA. MS analyses were performed on an Orbitrap Fusion Lumos Tribrid Mass Spectrometer (Thermo Fisher Scientific). LC conditions (instrumentation, column and gradient) were the same as described above.

Survey scans were performed at 15,000 resolution, with a scan range of 350–1,500 *m*/*z*, maximum injection time 50 ms and AGC target 4.5 × 10^5^. MS/MS DIA was performed in the orbitrap at 30,000 resolution with a scan range of 200–2,000 *m*/*z*. The mass range was set to ‘normal’, the maximum injection time to 54 ms and the AGC target to 2.0 × 10^5^. The inclusion mass list with the corresponding isolation windows is shown in Table 3. Data for both survey and MS/MS scans were acquired in profile mode. A blank sample (0.1% TFA, 80% MeCN, 1:1 v:v) was run between each sample to avoid carryover.

**Table 3:** List of masses, default charge states and isolation windows used for data independent acquisition

*m*/*z*	z	Time start (min)	Time stop (min)	Isolation window (*m*/*z*)
410	3	0	155	20
430	3	0	155	20
450	3	0	155	20
470	3	0	155	20
490	3	0	155	20
510	3	0	155	20
530	3	0	155	20
550	3	0	155	20
570	3	0	155	20
590	3	0	155	20
610	3	0	155	20
630	3	0	155	20
650	3	0	155	20
670	3	0	155	20
690	3	0	155	20
710	3	0	155	20
730	3	0	155	20
750	3	0	155	20
770	3	0	155	20
790	3	0	155	20
820	3	0	155	40
860	3	0	155	40
910	3	0	155	60
970	3	0	155	60

MS raw data files were processed using Spectronaught v.14.7.201007.47784 with either a human or a mouse reference FASTA sequence from UniProt, using default search parameters. The resulting protein-level data were analyzed using R v.3.5.0. Protein parts per million (p.p.m.) intensities were calculated by dividing the mean p.p.m. intensities between conditions (for example, for the human monocyte samples, ARDS patients and healthy controls), the *P* values were calculated using a Student’s *t*-test on log(transformed p.p.m. intensities). Proteins were designated as significantly changing if they showed *P* values <0.05 and fold-changes exceeding 1.96 s.d. away from the mean (that is, *z*-score >1.96). Only proteins that were quantified in all samples are shown in the volcano plot.

#### Gene expression analysis

Normalization of data was carried out using the geNorm selection of housekeeping genes function on NanoString nCounter analysis software. The resulting log_2_(normalized values) were used in subsequent analyses. Differential genes (‘DE genes’) were defined as genes with log_2_(fold-change) > 1, *P* < 0.05 across sample groups. Hierarchical clustering of sets of DE genes was carried out using Euclidian and Ward methods based on Pearson’s correlation values across transcriptional scores. The *z*-score scalar normalization of data was applied to the data before plotting as heatmaps. Analyses, including the drawing of heatmaps and volcano plots, were carried out in R using the package ggplot2 (https://cran.r-project.org/web/packages/ggplot2/index.html). Analysis of datasets was carried out by Thomson Bioinformatics, Edinburgh, UK.

#### Quantification, statistical analysis and reproducibility

Statistical tests were performed using Prism 8.00 and 9.0.2 software (GraphPad Software Inc.) (specific tests detailed in figure legends). Significance was defined as a *P* < 0.05 (after correction for multiple comparisons where applicable). Sample sizes (with each *n* number representing a different blood donor for human cells or an individual mouse for animal experiments) are shown in each figure.

### Reporting Summary

Further information on research design is available in the Nature Research Reporting Summary linked to this article.

## Online content

Any methods, additional references, Nature Research reporting summaries, extended data, supplementary information, acknowledgements, peer review information; details of author contributions and competing interests; and statements of data and code availability are available at 10.1038/s41590-022-01216-z.

## Supplementary information

Supplementary Information Supplementary Table 1.

Reporting Summary

Peer Review File

## Data Availability

The present study did not generate new unique reagents. All NanoString data shown in this manuscript have been deposited in the Gene Expression Omnibus (GEO) at accession nos.: GSE200429, GSE200549, GSE200558. All proteomic data generated in this project have been deposited in Pride at accession nos.: PXD033151. Analyses, including the drawing of heatmaps and volcano plots, were carried out in R using the package ggplot2 (https://cran.r-project.org/web/packages/ggplot2/index.html). Analysis of datasets was carried out by Thomson Bioinformatics, Edinburgh, UK.

## Extended data

Extended data are available for this paper at 10.1038/s41590-022-01216-z.
