# Author Correction: Hypoxia shapes the immune landscape in lung injury and promotes the persistence of inflammation

**DOI:** 10.1038/s41590-022-01286-z

**Published:** 2022-07-19

**Authors:** Ananda S. Mirchandani, Stephen J. Jenkins, Calum C. Bain, Manuel A. Sanchez-Garcia, Hannah Lawson, Patricia Coelho, Fiona Murphy, David M. Griffith, Ailiang Zhang, Tyler Morrison, Tony Ly, Simone Arienti, Pranvera Sadiku, Emily R. Watts, Rebecca. S. Dickinson, Leila Reyes, George Cooper, Sarah Clark, David Lewis, Van Kelly, Christos Spanos, Kathryn M. Musgrave, Liam Delaney, Isla Harper, Jonathan Scott, Nicholas J. Parkinson, Anthony J. Rostron, J. Kenneth Baillie, Sara Clohisey, Clare Pridans, Lara Campana, Philip Starkey Lewis, A. John Simpson, David H. Dockrell, Jürgen Schwarze, Nikhil Hirani, Peter J. Ratcliffe, Christopher W. Pugh, Kamil Kranc, Stuart J. Forbes, Moira K. B. Whyte, Sarah R. Walmsley

**Affiliations:** 1 University of Edinburgh Centre for Inflammation Research, Queen’s Medical Research Institute, University of Edinburgh, Edinburgh, UK; 2 Barts Cancer Institute, Queen Mary University of London, London, UK; 3 Wellcome Centre for Cell Biology, School of Biological Sciences, University of Edinburgh, Edinburgh, UK; 4 Intensive Care Unit, Royal Infirmary of Edinburgh, NHS Lothian, Edinburgh, UK; 5 Translational and Clinical Research Institute, Newcastle University, Newcastle upon Tyne, UK; 6 Department of Respiratory Medicine, Newcastle upon Tyne Hospitals NHS Foundation Trust, Newcastle upon Tyne, UK; 7 Roslin Institute, University of Edinburgh, Edinburgh, UK; 8 Centre for Regenerative Medicine, University of Edinburgh, Edinburgh, UK; 9 Nuffield Department of Medicine Research Building, Nuffield Department of Medicine, University of Oxford, Oxford, UK; 10 Ludwig Institute for Cancer Research, Nuffield Department of Medicine, University of Oxford, Oxford, UK; 11 The Francis Crick Institute, London, UK

**Keywords:** Monocytes and macrophages, Acute inflammation, Mucosal immunology, Haematopoiesis

Correction to: *Nature Immunology* 10.1038/s41590-022-01216-z, published online 27 May 2022.

In the version of this article originally published, in the Methods section “Mouse LPS ALI model,” the second sentence needed clarification of wording and dosage (mg kg^–1^, not mg g^–1^) and has been amended to read “Mice were treated daily (days 1–4 post-LPS), by subcutaneous injection, with PBS or 0.75 mg kg^–1^ of porcine CSF-1 fused to the Fc region of porcine IgG1a (generated by David Hume), prior to cull on day 5” in the HTML and PDF versions of the article.

