# Supplementary material for: Hypoxia shapes the immune landscape in lung injury and promotes the persistence of inflammation
Source: Nat Immunol. 2022 May 27;23(6):927–39. doi: 10.1038/s41590-022-01216-z (PMC9174051; doi:10.1038/s41590-022-01216-z)
Supplement: Supplementary file 1 [file 41590_2022_1216_MOESM1_ESM.pdf]

nature portfolio

https://doi.org/10.1038/s41590-022-01216-z

Supplementary information

Hypoxia shapes the immune landscape in lung injury and promotes the persistence of inflammation

In the format provided by the authors and unedited

Adam8	*Il17Ra*	*Malt1*	*Ptafr*
Anxa1	*Il6Ra*	*Man2b1*	*Ptgs1*
Cd99	*Itga5*	*Map2k4*	*Rab3d*
Eifebp2	*Lat2*	*Ncf2*	*Sema4a*
H2-DMb2	*Ltb4r1*	*Nlrp3*	*H2-Dma*

Supplementary Table 1: CSF1 upregulates genes that were downregulated in human ARDS monocytes

Differentially downregulated genes found in ARDS patients versus healthy control were compared to differentially upregulated genes found in mouse classical monocytes from hypoxic LPS-challenged CSF1-Fc-treated mice, relative to their PBS controls.
