# Supplementary material for: Hypoxia shapes the immune landscape in lung injury and promotes the persistence of inflammation
Source: Nat Immunol. 2022 May 27;23(6):927–39. doi: 10.1038/s41590-022-01216-z (PMC9174051; doi:10.1038/s41590-022-01216-z)
Supplement: Supplementary file 2 [file 41590_2022_1216_MOESM2_ESM.pdf]

Corresponding author(s): Ananda Mirchandani

Last updated by author(s): Apr 8, 2022

Reporting Summary

Nature Research wishes to improve the reproducibility of the work that we publish. This form provides structure for consistency and transparency in reporting. For further information on Nature Research policies, see our Editorial Policies and the Editorial Policy Checklist.

Statistics

For all statistical analyses, confirm that the following items are present in the figure legend, table legend, main text, or Methods section.

n/a	Confirmed	
☐	☒	The exact sample size (n) for each experimental group/condition, given as a discrete number and unit of measurement
☐	☒	A statement on whether measurements were taken from distinct samples or whether the same sample was measured repeatedly
☐	☒	The statistical test(s) used AND whether they are one- or two-sided<br>*Only common tests should be described solely by name; describe more complex techniques in the Methods section.*
☒	☐	A description of all covariates tested
☐	☒	A description of any assumptions or corrections, such as tests of normality and adjustment for multiple comparisons
☐	☒	A full description of the statistical parameters including central tendency (e.g. means) or other basic estimates (e.g. regression coefficient) AND variation (e.g. standard deviation) or associated estimates of uncertainty (e.g. confidence intervals)
☐	☒	For null hypothesis testing, the test statistic (e.g. F, t, r) with confidence intervals, effect sizes, degrees of freedom and P value noted<br>*Give P values as exact values whenever suitable.*
☒	☐	For Bayesian analysis, information on the choice of priors and Markov chain Monte Carlo settings
☒	☐	For hierarchical and complex designs, identification of the appropriate level for tests and full reporting of outcomes
☐	☒	Estimates of effect sizes (e.g. Cohen's d, Pearson's r), indicating how they were calculated

Our web collection on statistics for biologists contains articles on many of the points above.

Software and code

Policy information about availability of computer code

Data collection	No code was generated
Data analysis	Analyses, including the drawing of heatmaps and volcano plots were carried out in R using the package ggplot2 (https://cran.r-project.org/web/packages/ggplot2/index.html)<br>Data was analysed with Graphpad prism (8-9.0.2) and Treestar Flowjo (10.7.1)<br>MS raw data files were processed using Spectronaut v14.7.201007.47784 using a human reference FASTA sequence from UniProt using default search parameters. The resulting protein-level data were analysed using R v3.5.0.<br>BD FACSDiva 8 (multiple interations), FACSDiva 9.0 and FACSDiva 9.0.1. were used to acquire the data.

For manuscripts utilizing custom algorithms or software that are central to the research but not yet described in published literature, software must be made available to editors and reviewers. We strongly encourage code deposition in a community repository (e.g. GitHub). See the Nature Research guidelines for submitting code & software for further information.

Data

Policy information about availability of data

All manuscripts must include a data availability statement. This statement should provide the following information, where applicable:
- Accession codes, unique identifiers, or web links for publicly available datasets
- A list of figures that have associated raw data
- A description of any restrictions on data availability

All nanostring and proteomic data have been uploaded to Geo and Pride respectively. Accession codes to follow.

Field-specific reporting

Please select the one below that is the best fit for your research. If you are not sure, read the appropriate sections before making your selection.

☒ Life sciences ☐ Behavioural & social sciences ☐ Ecological, evolutionary & environmental sciences

For a reference copy of the document with all sections, see nature.com/documents/nr-reporting-summary-flat.pdf

Life sciences study design

All studies must disclose on these points even when the disclosure is negative.

Sample size	No sample calculations were performed pre-hoc. Groups of at least 3 were used in each independent experiment in treatment groups. Data from individual replicate experiments were pooled as detailed in the figure legends.No sample calculations were performed pre-hoc. Sample size was based on pilot experiments and previous experience with similar experiments in the laboratory. Groups of at least 3 mice per group were used in independent experiments. Data from individual experiments were pooled (as detailed in figure legends) to achieve power.
Data exclusions	In Fig Sb IFNb elisa one sample was excluded as found to be an outlier by ROUT testing on Graphpad Prism.
Replication	Experiments were replicated and pooled as detailed in the figure legends.
Randomization	In hypoxic studies, mice were randomly allocated to normoxic and hypoxic groups. Mixing of cages was not permitted where male mice mice were used but were purchased as part of the same cohort. Mice were age- and sex-matched within and between experiments.
Blinding	Investigators were not blinded during data collection if the obtained data were not subjective mainly due to the fact that blinding is complex when maintaining mice in hypoxic conditions. Analysis of immunofluorescence was performed blinded.

Reporting for specific materials, systems and methods

We require information from authors about some types of materials, experimental systems and methods used in many studies. Here, indicate whether each material, system or method listed is relevant to your study. If you are not sure if a list item applies to your research, read the appropriate section before selecting a response.

Materials & experimental systems

n/a	Involved in the study	
☐	☒	Antibodies
☒	☐	Eukaryotic cell lines
☒	☐	Palaeontology and archaeology
☐	☒	Animals and other organisms
☐	☒	Human research participants
☒	☐	Clinical data
☒	☐	Dual use research of concern

Methods

n/a	Involved in the study	
☒	☐	ChIP-seq
☐	☒	Flow cytometry
☒	☐	MRI-based neuroimaging

Antibodies

Antibodies used

CD16 eBioCD16 1-9161-71 4304474 FITC Ebioscience
CD3 OKT3 317308 B256076 PE Biolegend
CD56 HCD56 318306 B252053 PE Biolegend
CD19 HIB19 302254 B227178 PE Biolegend
CCR2 K036C2 357212 B260108 PE/Cy7 Biolegend
ICAM HCD54 322718 B193832 AF 647 Biolegend
CD45 2D1 368514 B248834 AF 700 Biolegend
CD14 M5E2 301820 B274258 APC/Cy7 Biolegend
HLA-DR L243 307624 B278326 Pacific Blue Biolegend
CD66b G10F5 305106 B278603 PE Biolegend
SiglecF E50-2440 552126 7058859 PE BD Biosciences
CD11b M1/70 101256 B238075 PE Dazzle BD Biosciences
CD11b M1/70 101243 B253527 BV785 Biolegend
MHCII M5.114.15.2 107624 B267551 PerCP Ebioscience
Epcam G8.8 118230 B251914 APCCy7 Fire Biolegend
CD3 17A2 100244 B198733 BIOTIN Biolegend
CD3 17A2 100213 B261416 Pacific Blue Biolegend
CD3 17A2 100229 B282101 BV 650 Biolegend
CD3 17A2 100206 B210714 PE Biolegend

CD19 6D5 115541 B242632 BV650 Biolegend
CD19 6D5 115504 B244881 Biotin Biolegend
CD19 6D5 115526 B265435 Pacific Blue Biolegend
CD19 6D5 115508 B223615 PE Biolegend
CD103 2E7 121433 BV605 Biolegend
Ly6G 1A8 127604 B218526 BIOTIN Biolegend
Ly6G 1A8 127608 B221647 PE Biolegend
Ly6G 1A8 127628 B280589 BV 421 Biolegend
Ly6G 1A8 135512 B213676 AF 488 Biolegend
Lyve-1 ALY7 50-0443-82 2205461 eFluor 660 Ebioscience
CD115 AFS98 135510 B211309 APC Biolegend
CD115 AFS98 128006 B217035 FITC Biolegend
Ly6C HK1.4 128032 B232012 BV 421 Biolegend
Pan-CD45 30-F11 103128 B274307 AF 700 Biolegend
CD11c N418 117318 B222652 PE/Cy7 Biolegend
CD11c N418 117352 B218048 APC/Fire750 Biolegend
CD64 X54-5/7.1 139304 B191540 PE Biolegend
CD64 X54-5/7.1 139306 B207411 APC Biolegend
CD4 H129.19 553649 Biotin BD Biosciences
CD5 53-7.3 553019 Biotin BD Biosciences
CD5 53-7.3 100603 B254317 Biotin Biolegend
CD8a 53 -6.7 553029 Biotin BD Biosciences
CD11b M1/70 101256 B238075 Biotin BD Biosciences
CD45R/B220 RA3-6B2 553086 Biotin BD Biosciences
Ter119
TER-119 116204
B295203 Biotin Biolegend
Ter119

TER-119

553672

Biotin

BD Biosciences

Gr-1/Ly-6G/C RB6-8C5 553125 Biotin BD Biosciences
CD117/cKit 2B8 105811 B249345 APC Biolegend
Sca-1/Ly- E13-161.7 122506 FITC Biolegend
CD48 HM48-1 103406 PE Biolegend
CD150 12F12.2 115914 PECy7 Biolegend
CD71 RI7217 113807 PE Biolegend
FcBlock CD16/32 93 101320 B295040 Biolegend
Streptavidin - 405232 B251688 BV 650 Biolegend
Streptavidin - Pacific Blue BD biosciences
LIVE/DEAD® Fixable Aqua - L34957
2068285 UV650 Life Technologies or Biolegend
CD45.2 104 109822 B252126 AF700 Biolegend
CD45.1 A20 110741 B253101 BV510 Biolegend
Sca.1 D7 108129 B262926 BV510 Biolegend
CD150 TC15-12F12.2 115903 PE Biolegend
CD105 MJ7/18 120412 B245562 PacBlue Biolegend
CD41 MWReg30 133927 B268849 APCCy7 Biolegend
IFNAR MAR1-5A3 127325 B286788 PECy7 Biolegend
CD5 53-7.3 100603 B254317 Biotin Biolegend
Ly6G 1A8 127604 B218529 Biotin Biolegend
B220 RA3-6B2 103204 B288658 Biotin Biolegend
CD11b M1/70 562287 CF594 Biolegend
F480 CI:A3-1 ab6640 Purified Abcam
IL-10 JES5-2A5 ab189392 Purified Abcam
LYVE-1 Polyclonal 103-PA50AG Purified ReliaTech GmbH
HIF-1 alpha Polyclonal NB100-479 Purified Novus Biotech
CD11b
M1/70 101243 B287244 BV 785 Biolegend

Validation

No primary antibodies were used in this study and the commercially-used antibodies used were used as per manufacturer's guidance

Animals and other organisms

Policy information about studies involving animals; ARRIVE guidelines recommended for reporting animal research

Laboratory animals

All animal experiments were performed under a UK Home Office-apporoved animal license held by S.R.W. (PPL 70/8364) and local

Laboratory animals	ethical approval. Wild type C57BL/6J male and female mice, aged 7 weeks to 3 months were used. Cd45.1+Cd45.2+, Cd45.2+/+ and Ifnar1 -/- mice were bred within the University of Edinburgh. Mice were held in IVC cages prior to use and where possible, were housed in the same room as where the hypoxic chamber was held. This was not possible in live infection experiments. Age- and sex-matched C57BL/6J mice were used as controls for Ifnar1 experiments following a period of acclimatization. Mice were kept in normal 12 hour day/ night cycles, in ambient temperature of 21 degrees celscius.
Wild animals	nil
Field-collected samples	nil
Ethics oversight	all animal experiments were performed by reseachers holding a personal animal licence and under the approval of a Home Office Animal Licence held by S.R.W. PPL 70/8364 and local ethical approval of each study by the University of Edinburgh BVS Unit veterinary team

Note that full information on the approval of the study protocol must also be provided in the manuscript.

Human research participants

Policy information about studies involving human research participants

Population characteristics	Blood was obtained from patients admitted to the Intensive Care Unit of Edinburgh Royal Infirmary who were diagnosed by the clinical team as having ARDS as per Berlin classification. Exclusion criteria included underlying malignancy, bone marrow disease or immunosuppression. Patient characteristics were as per supplementary table 1. Healthy control blood was obtained with ethical approval (see methods) with M:F 10:11 and median age 42 (IQR 24.5)
Recruitment	The diagnosis of ARDS was solely performed by the clinical team in charge of the patient with no involvement of the research team. All sequential samples from patients identified based on the ethics inclusion criteria were processed between Feb 2018 and August 2020 were processed.
Ethics oversight	Patients with ARDS were recruited and informed consent obtained directly or by proxy under the “META-CYTE” study (17/SS/0136/AM01) and “ARDS-NEUT” study (20/SS/0002) as approved by the Scotland A Research Ethics Committee. Samples were also obtained under the “Effects of Critical Illness on the Innate Immune System" study as approved by Health Research Authority (REC number 18/NE/0036).<br>All healthy participants gave written informed consent in accordance with the Declaration of Helsinki principles, with AMREC approval for the study of healthy human volunteers through the MRC / University of Edinburgh Centre for Inflammation Research blood resource (15-HV-013).

Note that full information on the approval of the study protocol must also be provided in the manuscript.

Flow Cytometry

Plots

Confirm that:

☒ The axis labels state the marker and fluorochrome used (e.g. CD4-FITC).

☒ The axis scales are clearly visible. Include numbers along axes only for bottom left plot of group (a 'group' is an analysis of identical markers).

☒ All plots are contour plots with outliers or pseudocolor plots.

☒ A numerical value for number of cells or percentage (with statistics) is provided.

Methodology

Sample preparation	Lung and alveolar cell sampling<br>Mice were culled with an overdose of intraperitoneal anesthetic (Euthetal) followed by blood collection from the inferior vena cava. Alveolar leukocytes were collected by bronchoalveolar lavage (BAL), then mice were perfused lightly with PBS through the heart, prior to harvest of lung tissue. On occasion, lower limbs were harvested for bone marrow leukocyte assessment (see below).<br>Tissue leukocytes were extracted from surgically dissociated lung tissue by enzymatic digestion with 2ml enzyme mix (RPMI with 0.625 mg/ml collagenase D (Roche), 0.85 mg/ml collagenase V (Sigma-Aldrich), 1 mg/ml dispase (Gibco, Invitrogen) and 30 U/ml DNase (Roche Diagnostics GmbH) for 45 minutes at 37C in a shaking incubator. The digest material was passed through a 100µM cell strainer with the addition of FACS buffer (PBS with 0.5% BSA/2% fetal calf serum and 0.02mM EDTA). Cell pellets were treated with red cell lysis buffer (Sigma) and washed in FACS buffer. The resulting cell suspension was subsequently passed through a 40µm strainer before cell counting using a Casey TT counter (Roche)/Nucleocounter or BioRad counter. Single cell suspensions (5 million cells/sample) were then stained for flow cytometry. BAL samples were counted prior to staining for flow cytometry.<br>Blood and bone marrow sampling<br>Mouse blood and bone marrow were treated with red blood cell lysis buffer (Biolegend) prior to counting and staining for flow cytometry (see Table 1).<br>Hematopoietic cell assessment was performed using both hind legs that were crushed using a pestle and mortar until a homogenous cell suspension was achieved or flushed though using a 32G needle. Cells were collected in cold FACS buffer and filtered through a 70 um nylon strainer (BD Falcon, 352340). Cells were treated with RBC lysis buffer (Biolegend) prior to

	staining. Where CD16/32 was assessed, this Ab was used prior to Fc-blocking
Instrument	Data was acquired using an LSR Fortessa (BD) and cells were sorted with an Aria II (BD) or Fusion (BD)
Software	Data was analyzed in FlowJo version 10 or FCS Express 7 for tsne analysis
Cell population abundance	Cell abundance as per each panel. For nanostring experiments, 5000 classical monocytes (mouse) or monocytes (human) or lung macrophages (mouse) were sorted directly into RLT buffer for subsequent analysis. For classical monocyte
Gating strategy	Human monocytes: Singles Dapi-CD45+non-granulocyte Lin(CD3/CD56/CD19/CD66b)- HLADR+ CD14+ and/or CD16+ cells<br>Mouse blood monocytes: Singles Dapi-CD45+ Lin(CD3/CD19/ Ly6G)-CD115+CD11b+Ly6Chi, Ly6Cint or Ly6C-<br>Mouse blood neutrophils: Singles, Dapi-CD45+Ly6G+CD11b+Ly6C int<br>Mouse lung/ BAL alveolar macrophages: Singles, Zombie Aqua-CD45+Lin (CD3/CD19/Ly6G)-CD64+SiglecF+CD11c+<br>Mouse lung interstitial / BAL inflammatory macrophages: Singles, Zombie Aqua-CD45+Lin (CD3/CD19/Ly6G)-CD64+SiglecF-CD11c+/- then Ly6C+/- MHCII+/-<br>Lung monocytes: Singles, Zombie Aqua-SinglesCD45+Lin (CD3/CD19/Ly6G)-CD64int CD11b+ Ly6C+<br>Lung/ BAL neutrophils: Singles Aqua or Dapi-, CD45+CD11b+Ly6G+<br>Lung cDC1 Zombie Aqua-Singles CD45+CD11chi,CD103+, CD64-MHCII+<br>BM HSPC SLAM analysis Alive, Singles LK (Lin-cKit+) and LSK (Lin-cKit+Sca-1+) cells. LSK cells were further sub-gated on hematopoietic stem cells (HSCs: LSK CD48-CD150+), multipotent progenitors (MPPs: LSK CD48-CD150-), hematopoietic progenitor cells-1 (HPC-1: LSK CD48+CD150-) and hematopoietic progenitor cells-2 (HPC-2: LSK CD48+CD150+)<br>BM erythroid progenitors based on Pronk analysis 26 Singles, Dapi or Aqua-, Lin-, CD11b-, cKit+, Sca1-, CD32/16-, CD41-, CD105+ or CD150+ (Pre-MegE CD150+CD105-, Pre-CFUE CD150+CD105+, CFUE CD150-CD105+)<br>Further gating strategy information can be made available upon request.

☒ Tick this box to confirm that a figure exemplifying the gating strategy is provided in the Supplementary Information.
